# Prevalence, incidence, and risk factors associated with cytomegalovirus infection in healthcare and childcare worker: a systematic review and meta-analysis

**DOI:** 10.1186/s13643-022-02004-4

**Published:** 2022-06-27

**Authors:** Safari Joseph Balegamire, Elisabeth McClymont, Agathe Croteau, Philippe Dodin, Soren Gantt, Amir Abbas Besharati, Christian Renaud, Benoît Mâsse, Isabelle Boucoiran

**Affiliations:** 1 Department of Social and Preventive Medicine, École de Santé Publique de Université de Montréal, Montreal, QC Canada; 2 Women and Children’s Infectious Diseases Center, CHU Sainte-Justine Research Center, Montreal, QC Canada; 3 Department of Pediatrics, University of British Columbia, Vancouver, BC Canada; 4 National Institute of Public Health of Québec, Québec City, QC Canada; 5 Department of Microbiology, CHU Sainte-Justine, Université de Montréal, Montréal, QC Canada; 6 Applied Clinical Research Unit, CHU Sainte Justine Research Center, Montreal, QC Canada; 7 Division of Maternofetal Medicine, Department of Obstetrics and Gynecology, Université de Montréal, Montreal, QC Canada

**Keywords:** Cytomegalovirus, Healthcare workers, Childcare, Day care, Prevalence, Young children, Incidence, Primary infection

## Abstract

**Background:**

Cytomegalovirus (CMV) is transmitted by direct contact with body fluids from infected individuals. Transmission of CMV in households, particularly those with young children, contributes significantly to CMV infection in the general population. However, little is known about the contribution of occupational healthcare or childcare exposure to risk of CMV infection.

**Objectives:**

To determine CMV seroprevalence, incidence of primary infection, and associated risk factors in healthcare and childcare workers.

**Methods:**

Six electronic databases were searched systematically for publications on CMV infection in healthcare and childcare workers until March 7, 2022. Two authors independently evaluated the literature for quality and inclusion in our analyses. The pooled results for seroprevalence, incidence, and relative risk (RR) were determined using a random effects model. Heterogeneity among studies was quantified and further investigated in subgroup analysis and meta-regression. Publication bias was assessed using funnel plot. Statistical analyses were preformed using R version 4.05.

**Results:**

Forty-eight articles were included in this meta-analysis (quality assessment: 18 good, 14 fair, and 16 poor). Pooled CMV seroprevalence was 59.3% (95% *CI*: 49.8–68.6) among childcare workers and 49.5% (95% *CI*: 40.3–58.7) among healthcare workers, and pooled incidences of primary CMV infection per 100 person-years were respectively 7.4 (95% *CI*: 3.9–11.8) and 3.1 (95% *CI*: 1.3–5.6). RR for primary infection compared to controls were 3.4 (95% *CI*: 1.3–8.8) and 1.3 (95% *CI*: 0.6–2.7) for healthcare and childcare workers, respectively. The odds of CMV seropositivity were 1.6 (95% *CI*: 1.2–2.3) times higher for childcare workers compared to controls, but not significantly different between healthcare workers and controls (0.9; 95% *CI*: 0.6–1.2). CMV seropositivity in both groups was significantly associated with having one or more children residing at home, marital status, ethnicity, and age.

**Conclusions:**

Childcare workers, but not healthcare workers, have an increased risk of prevalent and incident CMV infection, a risk that is further increased with the presence of at least one child living at home. These findings suggest that enforcing simple, conventional hygienic measures in childcare settings could help reduce transmission of CMV, and that special precautionary measures for preventing CMV infection may not be required for pregnant healthcare workers.

**Systematic review registration:**

PROSPERO CRD42020139756

**Supplementary Information:**

The online version contains supplementary material available at 10.1186/s13643-022-02004-4.

## Introduction

Cytomegalovirus (CMV) is a common infection with a seroprevalence ranging from 45 to 100% [1], depending on the population and existing risk factors [2–4].

CMV is a member of the herpesvirus family that characteristically produce latent and persistent infections in human hosts [5, 6]. CMV is transmitted horizontally through contact with biological fluids (saliva, urine, tears, semen, vaginal secretions, blood, and breast milk) from an infected individual or vertically (mother to fetus) by placental transfer [7]. CMV infection can be diagnosed directly with polymerase chain reaction (PCR), virus culture tests, and pp65 viral antigen detection or indirectly with serology and with IgG and IgM avidity tests [8].

CMV is the most common vertically transmitted infection, and congenital CMV infection is a major health concern as the leading cause of nongenetic hearing loss in children and its association with high rates of severe abnormal neurodevelopment [9–11].

Young children congenitally or postnatally infected with CMV, especially aged from 1 to 3 years, shed large amounts of virus in biological fluids over prolonged periods and represent important vectors of CMV transmission and infection [12–14]. Thus, exposure to young children in the workplace may predispose to the risk for CMV infection [15].

Due to the considerable global health burden of congenital CMV infection, exposure of women of childbearing age to CMV is of great interest to policy makers. To reduce the risk of CMV transmission/acquisition, certain countries, including Germany and parts of Canada, require that women exposed to young children in the workplace, namely healthcare and childcare workers, be reassigned or given leave of absence during pregnancy [16–18]. A meta-analysis studying CMV prevalence and risk of seropositivity and occupational exposure to children has recently been published that focused on childcare workers only and restricted the selection of studies to certain countries published beginning since the year 2000 [19]. The present study provides estimates of the prevalence and incidence of CMV, based on relevant studies from any country, whenever they were published, and identifies risk factors for seropositivity and primary infection in healthcare and childcare workers compared to respective control groups. Our findings reveal important differences between study groups and suggest ways to reduce the incidence of CMV infection.

## Methods

### Protocol design and registration

This is a systematic review and meta-analysis of prevalence, incidence, and risk factors associated with CMV infection in healthcare and childcare workers. PRISMA 2020 and MOOSE protocols were used as references for the search strategy and for reporting results (Additional file, Table S1) [20, 21]. The study protocol was registered under the International Prospective Register of Systematic Reviews—Prospero, number CRD42020139756.

### Study eligibility and selection

Two examiners (SJB, EM) blinded to one another independently evaluated all articles, and those pertaining to prevalence, to incidence, and to risk factors associated with CMV seropositivity were selected for inclusion (Table 1). Only studies in human published in French or English were considered. Non-original papers, case reports, and redundant articles were excluded. Any disagreements between article inclusion or exclusion were resolved by a third independent examiner (IB).

**Table 1:** Eligibility criteria

	Inclusion criteria	Exclusion criteria
Population	Employable	Age less than 16 or older than 80 years, animals
Exposure	Possible occupational exposure to CMV: childcare and healthcare workers	
Control	No occupational exposure to CMV: jobs not related to childcare or health care	
Outcomes	Ia. CMV seroprevalence Ib. Incidence of CMV primary infection IIa. Seroprevalence odds ratio IIb. Seroconversion risk ratio III. Risk factor odds ratio	
Study design	Any original study including more than one participant	Case report, comment, letter to the editor, ecological study, meta-analysis, systematic review

### Study outcomes

The main outcomes of interest were to estimate the prevalence, incidence, relative risks, and risk factors associated with CMV infection in healthcare and childcare workers.

### Search strategy

Systematic searches of the databases PubMed (NLM), Ovid MEDLINE, Ovid All EBM Reviews, Ovid Embase, ISI Web of Science, and EBSCO CINAHL Complete were performed by a trained librarian (PD) who retrieved all publications related to occupational exposure to CMV up until the cutoff date of March 7, 2022. The MeSH terms related to “cytomegalovirus” and “occupational exposure” were defined and combined for the search (Additional file, Table S2).

We also manually searched bibliographies from prior systematic reviews and meta-analyses, thoroughly reviewed articles cited in scientific reports, presentations of the experts from the “Institut National de Santé Publique de Québec,” and searched for articles in Google Scholar. Neither approach identified additional publications.

### Quality appraisal of included studies

The quality of included studies was evaluated using the NIH Study Quality Assessment Tools [22]. These tools were designed to help reviewers focus on concepts that are essential for critically assessing the internal validity of a study, but not to provide a list of factors that includes a numerical score; the guide to using this practical quality assessment tool is explained elsewhere [22]. A “good” quality study has the least risk of bias, and the results are considered valid. A study of “fair” quality is likely to have some risk of bias, but not enough to invalidate results. A study of “poor” quality indicates a high probability of risk of bias.

The quality of the studies included in the present review was assessed in a blinded manner by two independent examiners (SJB, EM). If opinions differed, an additional assessment was performed by a third examiner (IB), and a consensus was reached.

### Data extraction

Data were extracted independently by two examiners (SJB and EM) and compared to ensure accuracy. Data consisted of author, year of data collection, year of publication, article title, geographic location, risk factors for CMV, type of occupation, study setting, number of study sites, number of participants, and diagnostic method used for CMV. The populations of interest were healthcare and childcare workers, and the control groups represented participants with other jobs reported in the studies. The number of CMV seropositive cases and the number of cases that seroconverted were recorded, including, whenever possible, for the control populations. When relevant information was unavailable in the articles themselves, the authors were directly contacted.

### Statistical analysis

For each study included in the analyses, the prevalence of CMV was estimated in each group by dividing the number of seropositive cases by the total number of individuals tested. Similarly, the incidence of primary CMV infection expressed in person-years was calculated by dividing the number of individuals that seroconverted during a specified time frame by the total number of individuals tested during that time. The odds ratio (OR) for CMV seropositivity among healthcare and childcare workers was estimated by comparing their odds with those of the control groups. RR for primary CMV infection were calculated by dividing incidences in healthcare and childcare workers by the incidence in the respective control populations. Risk factors for CMV seropositivity were determined based on the odds of CMV seropositivity in each study group compared to these controls.

Statistical analyses were preformed using R version 4.05. A *p-*value less than 0.05 was considered statistically significant. Pooled prevalence and incidence estimates were obtained using the R *metapro* and *metarate* statistics packages [23] using a random effects model with a restricted maximum likelihood and Freeman-Tukey double-arcsine transformation to stabilize variances [24, 25].

Pooled OR and RR measures of CMV seropositivity and primary infection among healthcare and childcare workers versus controls were estimated using meta-analyses of binary outcome data (R *metabin*) [23, 26]. In the random effects model, variance between studies was estimated using the Paule and Mandel method [26]. To estimate the percentage of the total variation attributed to study heterogeneity, and not chance, we used the *I*^2^ statistic [27]; when *I*^2^ was greater than 40%, we studied the heterogeneity in subgroup and post-stratification analyses with the variables, occupational group (childcare workers versus healthcare staff), diagnostic method for CMV, study design, and study quality. Sensitivity analyses based on the study quality and sample size were performed. Meta-regression was employed to further explore study heterogeneity [28]. Funnel plot was used to assess publication bias, and Begg’s test rank correlation [29] or Egger’s linear regression method [30] was used to evaluate its asymmetry.

## Results

### Study characteristics

**Fig. 1:**
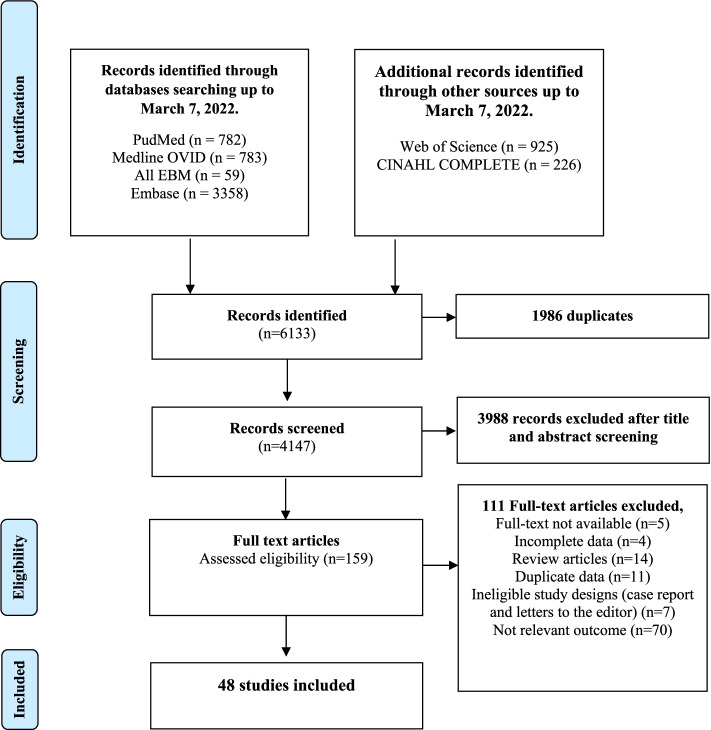
Flowchart describing the selection of studies on prevalence, incidence, and risk factors associated with CMV in childcare and healthcare workers

A PRISMA chart depicting the number of records identified, included, and excluded is provided in Fig. 1. Forty-eight articles (18 good quality, 14 fair quality, and 16 poor quality) were included in our analyses (Additional file, Table S3). From the 48 studies included in our analyses, 27 reported prevalence, 20 reported incidence and prevalence, and 1 reported on incidence only. Forty-seven studies were used to estimate pooled CMV seroprevalence, and 21 served to estimate the incidence of primary CMV infection (Additional file, Table S4 for a description of each article). A total of 29,486 healthcare and childcare workers from 16 countries (median: 183, range: 4–17,130 participants per study) were included in the analysis. Studies were primarily from the USA (44%), Canada (10%), France (8%), and Germany (6%); 35% were cohort studies, and 65% were cross-sectional studies. No experimental or clinical studies were identified that met our inclusion criteria. Forty-five percent (22/48) of the studies involved childcare workers, 50% (24/48) concerned healthcare workers, and two studies included both [31]. Methodologies used to assess CMV serostatus varied across studies and included ELIS (19/48), latex agglutination (6/48), complement fixation (7/48), anticomplement immunofluorescence (4/48), restriction endonuclease (2/48), unspecified (3/48), and methods defined as “other” (7/48).

### CMV seroprevalence and incidence of primary CMV infection among healthcare and childcare workers

**Fig. 2:**
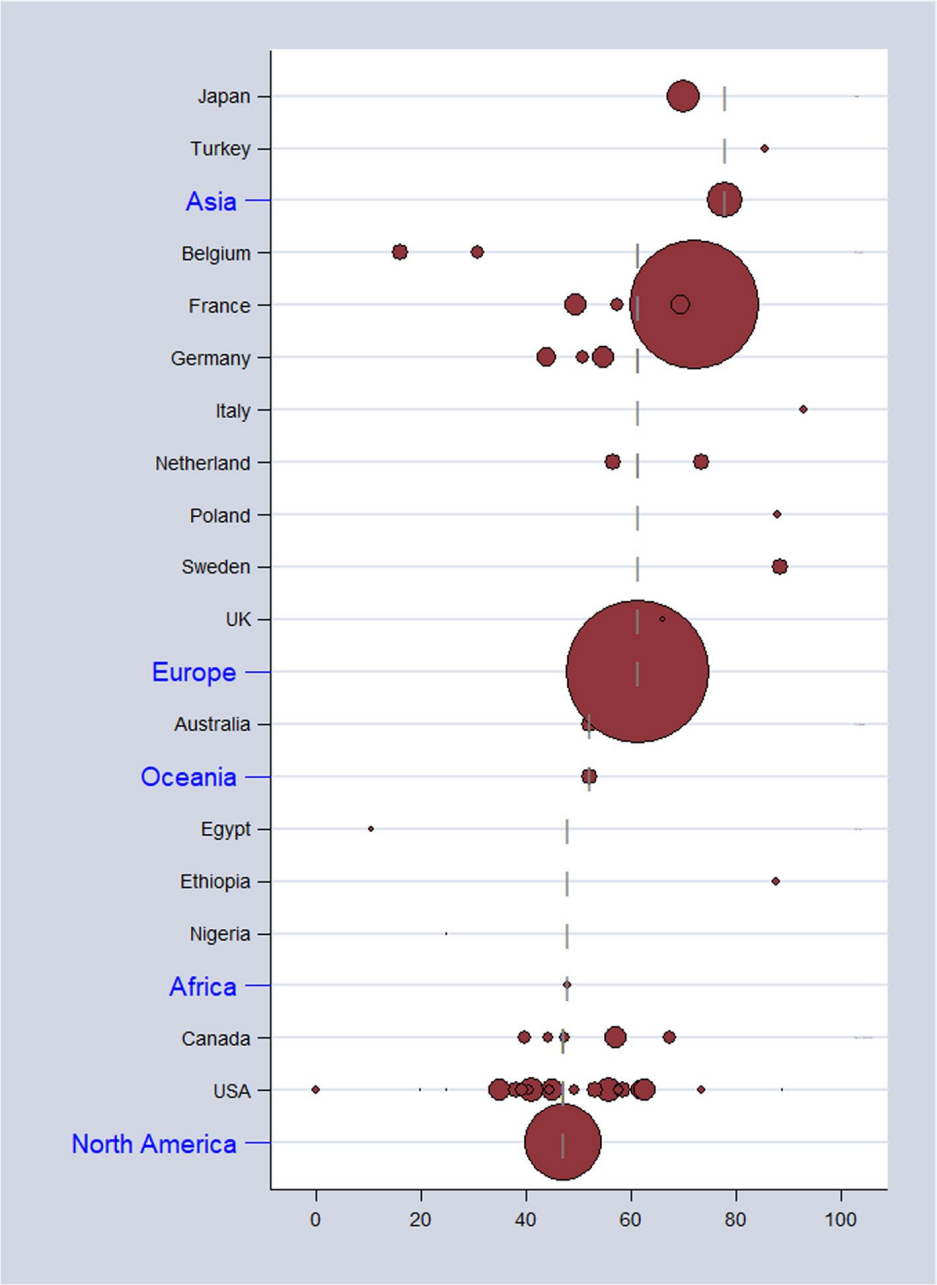
Estimates of seroprevalence of CMV infection by geographical location. Overall CMV seroprevalence 53.3% (95% *CI*: 46.5–60.0). Bubble plots of CMV pooled seroprevalence in populations exposed to children in the workplace. Individual study data are shown at the country level and pooled by continent. The size of bubble is proportional to the number of participants studied

**Fig. 3:**
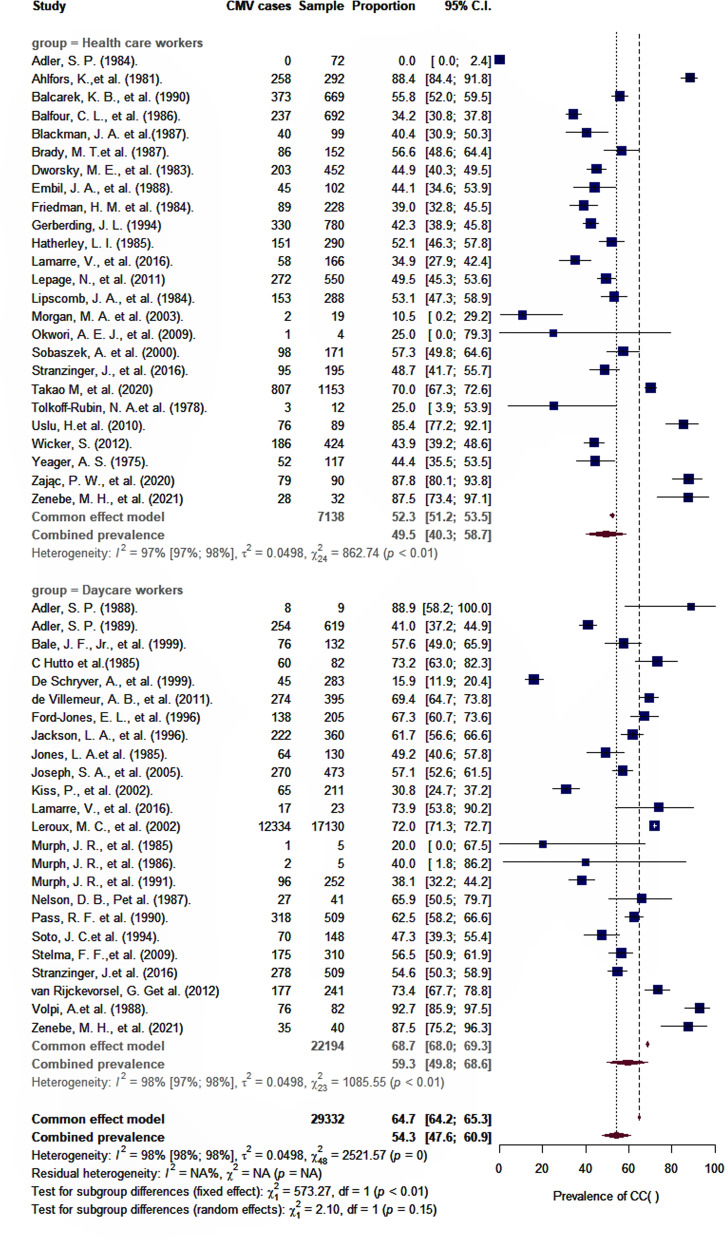
Pooled seroprevalence of CMV infection among childcare and healthcare workers

As shown in Table 2, the overall pooled seroprevalence among healthcare and childcare workers was 53.3% (95% *CI*: 46.5–60.0) and differed significantly (*p* < 0.0001) between continent (61% Europe, 78% Asia, 47% North America, 52% Oceania, and 48% Africa) (Fig. 2). When analyzed by subgroup, the seroprevalences among healthcare and childcare workers were 59.3% and 49.5%, respectively (Fig. 3), and these rates did not change significantly (59.2% and 54.6%) in the sensitivity analysis that excluded poor quality studies.

**Table 2:** Seroprevalence and incidence of CMV infection among healthcare and childcare workers

	CMV seropositivity				CMV primary infection			
Covariates	Number of studies	Sample size	Proportion % (95 % *CI*)	*I*^2^% (95% *CI*)	Number of studies	Person-year	Incidence % per year (95% *CI*)	*I*^2^ % (95% CI)
Overall prevalence	47	29332	53.3 (46.5–60.0)	98 (98–98)	21	3994	4.6 (2.6–7.1)	83 (76–89)
Groups*								
Childcare workers	24	22194	59.3 (49.8–68.6)	98 (97–98)	9	883	7.4 (3.9–11.8)	89 (81–93)
Healthcare workers	25	7138	49.5 (40.3–58.7)	97 (97–98)	12	3111	3.1 (1.3–5.6)	48 (0–73)
Region								
Africa	3	95	47.9 (18.5–78.0)	96 (91–98)	-			
North America	26	6781	46.6 (37.9–55.5)	94 (92–95)	15	3272	5.9 (3.3–9.0)	87 (80–91)
Asia	2	1242	77.9 (49.1–96.8)	91 (68–97)	1	70	2.8 (0.0–8.4)	-
Europe	15	20924	61.2 (50.2–71.7)	99 (98–99)	3	352	1.1 (0.0–7.4)	15 (0–91)
Oceania	1	290	52.1 (46.3–57.8)		2	300	2.3 (0.0–10.3)	62 (0–91)
Diagnostic method								
Anticomplement immunofluorescence	4	1466	60.5 (39.4–79.7)	97 (95–98)	3	1208	2.6 (0.1–7.3)	17 ( 0–91)
Complement fixation	7	1636	48.6 (31.4–65.9)	98 (97–99)	5	1178	4.4 (1.2–9.2)	72 (29–89)
ELISA	18	5245	53.1 (42.7–63.3)	98 (95–97)	5	1169	5.0 (1.8–9.5)	93 (88–97)
Latex agglutination	6	795	47.0 (28.9–65.5)	90 (81–95)	4	258	7.9 (2.8–15.1)	0 (0–85)
No specify	3	644	24.6 (7.1–48.0)	98 (97–99)	1	42	23.8 (12.0–38.0)	-
Other	7	19496	66.0 (50.4–80.1)	95 (92–97)	2	125	1.0 (0.0–7.1)	45 (–)
Restriction endonuclease	2	50	76.0 (41.3–98.8)	42 (–)	1	14	0.0 (0.0–11.9)	-
Study design								
Cohort	25	7548	52.1 (42.8–61.3)	97 (97–98)	21	3994	4.6 (2.6–7.1)	83 (76–89)
Cross-sectional	22	21784	54.7 (44.6–64.6)	98 (97–98)	-			
Quality								
Good	19	7584	58.9 (48.8–68.7)	98 (97–98)	10	2590	5.8 (2.7–9.8)	88 (79–93)
Fair	13	3160	51.1 (38.8–63.3)	96 (95–97)	8	1044	3.5 (0.7–7.8)	67 (30–84)
Poor	15	18588	46.7 (33.7–59.8)	98 (97–98)	3	359	3.9 (0.1–11.4)	92 (80–97)

*CI C*onfidence interval, *I*^2^Statistic for heterogeneity. *Two studies included childcare and healthcare workers

**Fig. 4:**
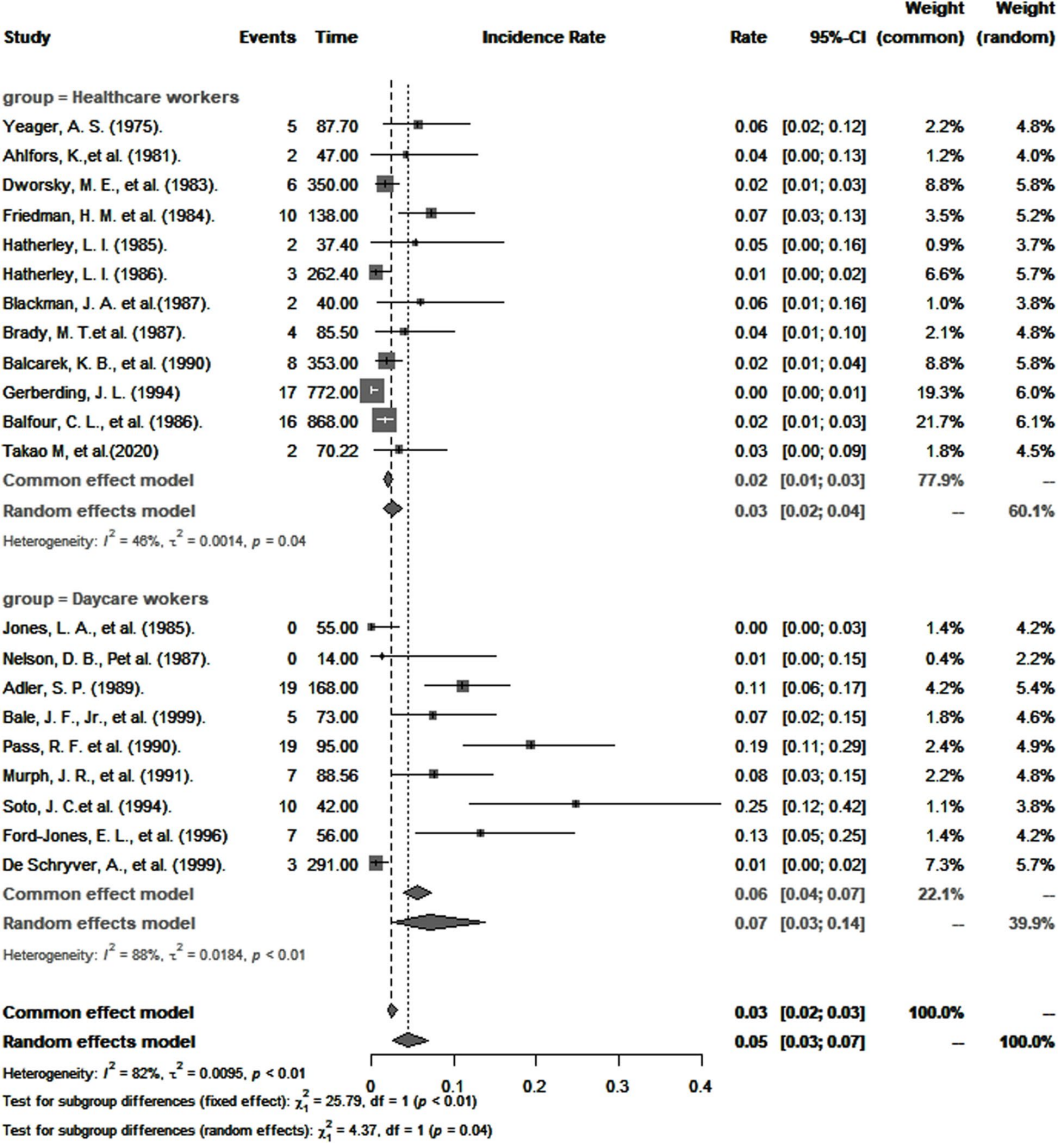
Pooled annual incidence of primary CMV infection

The overall pooled incidence of primary CMV infection among healthcare and childcare workers was 4.6 per 100 person-years (95% *CI*: 2.6–7.1). Results of subgroup analysis are presented in Table 2. Consistent with seroprevalence rates, the pooled annual incidence of primary CMV infection was statistically significantly higher among childcare workers than among healthcare workers (7.4 per 100 person-years versus 3.1 per 100 person-years; *p* < 0.0001; Fig. 4). A sensitivity analysis that omitted studies of poor quality did not significantly change these estimates (7.5 per 100 person-years versus 3.3 per 100 person-years).

### CMV seropositivity and primary infection among healthcare and childcare workers compared to control groups

**Fig. 5:**
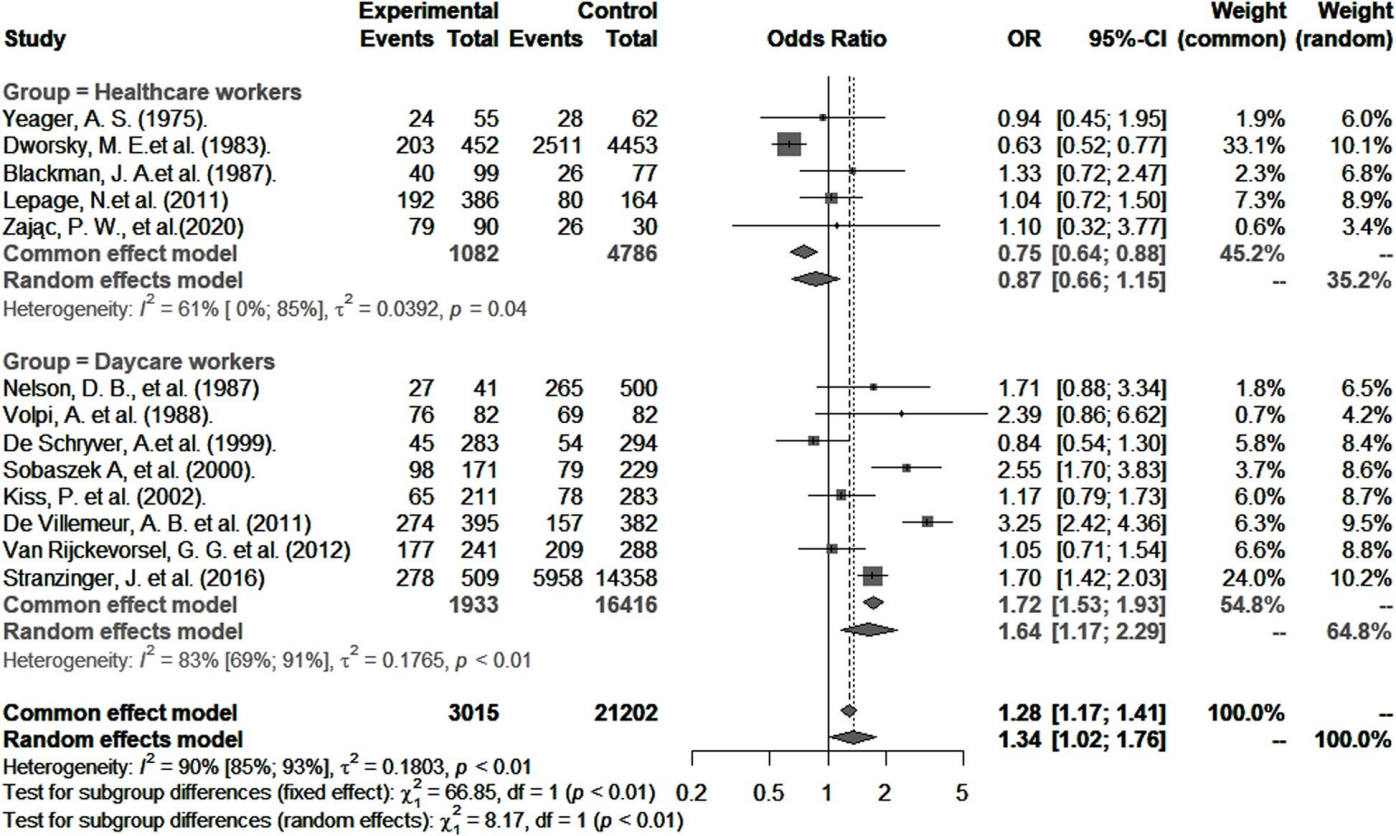
Risk of CMV seropositivity among healthcare and childcare workers compared to controls

**Fig. 6:**
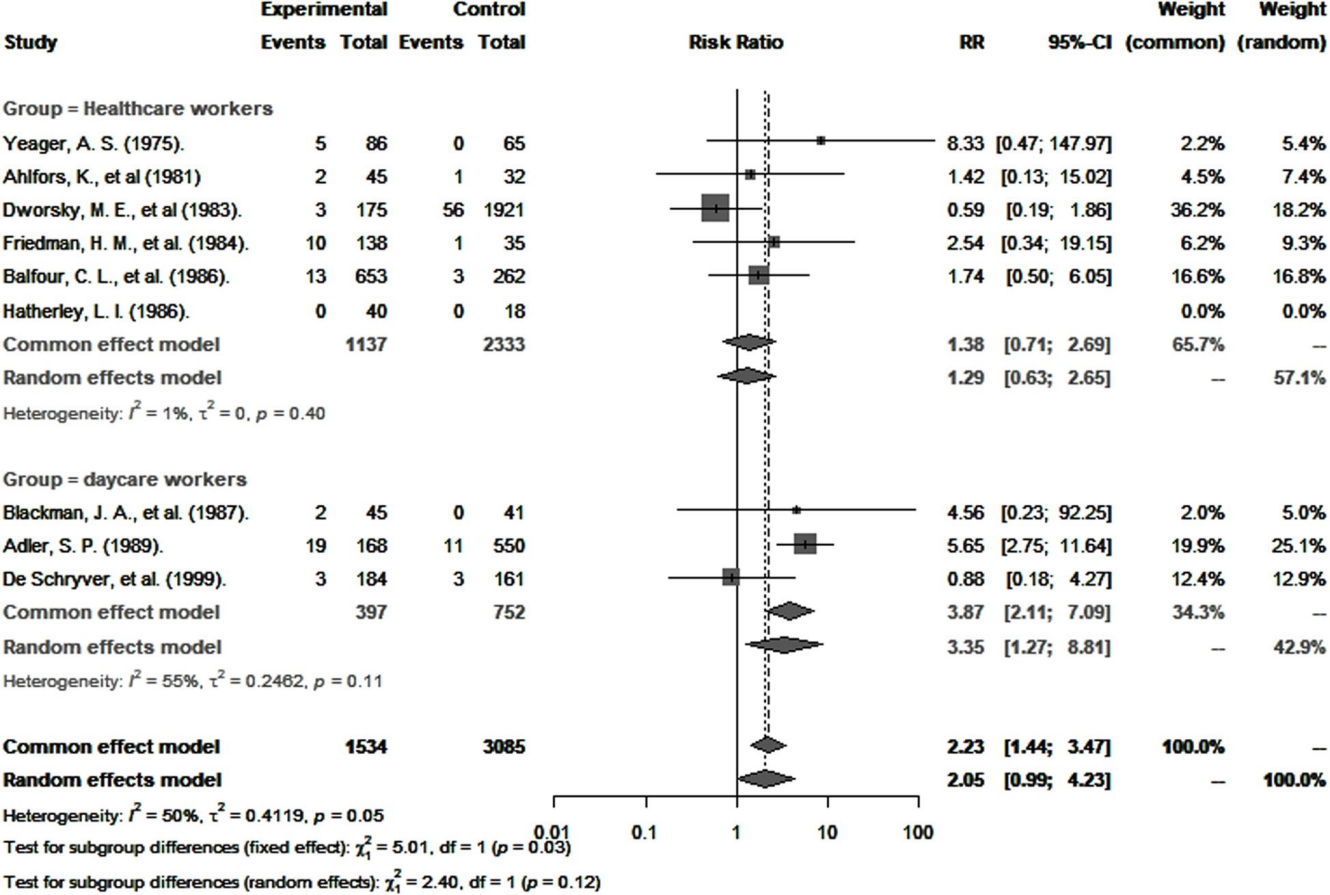
Risk ratio of primary CMV infection in healthcare and childcare workers compared to controls

CMV seropositivity and primary infection rates in healthcare and childcare workers were compared with those of controls (Table 3). The odds of CMV seropositivity were significantly higher among childcare workers (*OR* 1.6, 95% *CI*: 1.2–2.3), but not healthcare workers (*OR* 0.9, 95% *CI*: 0.6–1.2) (Fig. 5) when compared to their respective controls, and as shown in Fig. 6, so were the RR for CMV primary infection (3.4, 95% *CI*: 1.3–8.8 versus 1.3, 95% *CI*: 0.6–2.7) (Fig. 6). Sensitivity analysis that excluded poor quality studies had no significant impact on either the OR estimates for CMV seropositivity (*OR* 1.5; 95% *CI*: 1.1–2.2) versus (*OR* 0.9; 95% *CI*: 0.7–1.2, [*p* = 0.8530]) or RR estimates for primary infection (*RR* 3.4; 95% *CI*: 1.3–8.8) and (*RR* 1.3; 95% *CI*: 0.6–2.7, [*p* = 0.7425]) compared to respective controls.

**Table 3:** Comparison of CMV seropositivity and primary CMV infection rates in childcare and healthcare workers with controls

	Risk of CMV seropositivity					Risk of CMV primary infection				
Covariates	No. of studies	Exposed population	Nonexposed	OR (95% *CI*)	*I*^2^ % (95% *CI*)	No. of studies	Exposed population	Nonexposed	RR (95% *CI*)	*I*^2^ % (95% *CI*)
Overall	13	3015	21202	1.4 (1.01–1.80)	90 (85–93)	9	1534	3085	2.1 (1.0–4.2)	50 (0–78)
Groups										
Childcare workers	8	1933	16416	1.6 (1.2–2.3)	83 (69–91)	3	397	752	3.4 (1.3–8.8)	55 (0–87)
Healthcare workers	5	1082	4786	0.9 (0.6–1.2)	61 (0–85)	6	1137	2333	1.3 (0.6–2.7)	1 (0–80)
Continent										
America	3	606	4592	0.8 (0.5–1.3)	66 (0–90)	6	1265	2874	2.4 (1.1–5.1)	58 (0–83)
Europe	10	2409	16610	1.5 (1.1–2.1)	82 (68–90)	2	229	193	1.0 (0.3–3.8)	-
Oceania	-	-	-	-	-	1	40	18	-	
Diagnostic method										
Anticomplement immunofluorescence	2	534	4535	1.1 (0.3–4.0)	84 (35–96)	1	175	1921	0.6 (0.2–1.9)	-
Complement fixation	-	-	-	-	-	3	269	132	2.7 (0.7–10.6)	0 (0–76)
ELISA	6	1801	15616	1.3 (0.9–1.8)	79 (53–90)	4	1194	991	2.4 (0.8–7.2)	66 (0–90)
Latex agglutination	-	-	-	-	-	1	45	41	4.6 (0.2–92.5)	0
Other	5	680	1051	1.7 (1.1–2.7)	75 (39–90)					
Study design										
Cohort	5	930	5386	0.9 (0.7–1.3)	69 (20–88)					
Cross-sectional	8	2085	15816	1.6 (1.2–2.4)	82 (66–91)					
Quality										
Good	6	1378	1209	1.3 (0.9–2.0)	86 (73–93)	4	952	909	3.8 (1.9–7.6)	16 (0–87)
Fair	6	1466	19764	1.2 (0.8–1.8)	92 (85–95)	4	542	2158	0.9 (0.4–2.7)	0
Poor	1	171	229	2.6 (1.7–3.8)	-	1	40	18	-	-

*OR *Odds ratio, *RR *Risk ratio, *CI *Confidence interval, *I*^2^Statistic for heterogeneity

### Risk factors for CMV seropositivity among healthcare and childcare workers

Studies that categorized risk factors similarly were included in the pooled analyses of OR for CMV seropositivity (Table 4). In both groups, this OR was significantly higher in households with one or more children (childcare workers: *OR* 1.9; 95% *CI*: 1.3–2.7; healthcare workers: *OR* 2.2; 95% *CI*: 1.6–3.8). Other factors significantly associated with higher OR for CMV seropositivity were ethnicity other than Caucasian (*OR* 2.3; 95% *CI*: 1.7–3.1), marriage and common-law partnership (*OR* 1.7; 95% *CI*: 1.4–2.1), and, unique to healthcare workers, age ≥ 30 years (*OR* 2.6; 95% *CI*: 1.8–3.8).

**Table 4:** Odds ratio for CMV seropositivity by risk factor among childcare and healthcare workers

Covariates	Number of studies	Exposed population	Nonexposed	OR (95% *CI*)	*I*^2^ % (95% *CI*)
Children at home (yes vs no)	6	1000	947	2.0 (1.7–2.5)	6 (0–76)
Childcare workers	4	681	647	1.9 (1.3–2.7)	37 (0–78)
Healthcare workers	2	319	300	2.2 (1.6–3.8)	0
Race (others versus white)	3	283	1154	2.3 (1.7–3.1)	0 (0–89)
Marital status (married versus single)	4	1237	683	1.7 (1.4–2.1)	0 (0–70)
Age (30 years vs < 30 years)	7	1247	922	1.4 (0.8–2.6)	92 (86–95)
Childcare workers	5	1023	638	1.1 (0.5–2.3)	92 (85–96)
Healthcare workers	2	225	284	2.6 (1.8–3.8)	0

*OR *Odds ratio, *CI *Confidence interval, *I*^2^ Statistic for heterogeneity

### Publication bias and meta-regression

Funnel plot asymmetry analysis using the Begg’s and Egger’s tests did not reveal evidence of publication bias (Additional file, Table S5).

Multivariate meta-regression models were used to explore heterogeneity across studies (Additional file, Table S6). More specifically, they were employed to analyze heterogeneity in pooled analyses of CMV prevalence, incidence, risk of seropositivity, and primary CMV infection from non-stratified data. Multivariable meta-regression analysis was not possible to assess the source(s) of heterogeneity in risk factors due to a paucity of studies. We found that heterogeneity for CMV prevalence was largely attributed to studies that had not specified the diagnostic method for CMV, whereas heterogeneity in OR for CMV seropositivity could be explained by diagnostic method, study design, and study quality. Regarding incidence and RR for CMV primary infection, none of the variables tested were associated with heterogeneity.

## Discussion

This meta-analysis of 48 studies from 16 countries provides updated estimates for CMV seroprevalence, OR for seropositivity, and RR of primary infection in healthcare and childcare workers compared to their respective controls and extends our knowledge of the risk factors associated with CMV seropositivity in populations exposed to children in the workplace.

The results show that about half of all healthcare and childcare workers (53%) were seropositive for CMV. This estimate is considerably lower than worldwide (83%), European (70%), southeast Asian (89%), and African (89%) estimates [5] but comparable to rates estimated for the general adult population in the USA (50%) [32].

CMV transmission from infected children occurs primarily through direct inoculation of virus shed from body fluids, particularly saliva and urine, in host mucous membranes, and increased transmission appears to be associated with poor hygienic practices [33]. Compared to controls, our findings show a statistically significant increased odds for CMV seropositivity among childcare, but not healthcare workers, and are consistent with Starke et al. [34] who reported an increased risk of CMV infection in childcare workers compared to the general population and with Bale et al. [15] who showed that in addition to childcare workers, this risk was significantly greater among parents with children in childcare, compared with healthcare workers. This might be explained by better adherence to universal infection prevention protocols in healthcare versus childcare workplace settings [35]. It is worth noting that because healthcare workers are not uniformly exposed to the same risk of exposure to CMV, it is likely that their overall level of exposure was less than for childcare workers. Indeed, studies have shown that the prevalence and incidence rates of CMV infection are different in healthcare staff working in different hospital departments [36–39] depending on the nature of the patients they are exposed to.

The overall incidence of primary CMV infection from pooled studies of healthcare and childcare workers was 4.7 per 100 person-years and is within the range of estimates for pregnant women in the general population [40–42]. Among healthcare workers only, this incidence was 3.1 per 100 person-years and is comparable to that reported for nurses working in pediatric wards [43].

The significance of the higher risk of CMV primary infection observed in childcare workers compared to controls should be regarded with some caution because for controls, the risk was derived from the unadjusted analysis of only three studies, and major confounders, particularly age, socioeconomic status, and number of children living at home, were not considered. Nevertheless, that identical strains of CMV are observed in children attending daycare and in childcare workers [40, 44–46] suggest that the latter acquire it from the former. While the actual risk of congenital CMV infection in pregnant childcare workers has not been established, preventive measures and screening strategies should be implemented, especially for pregnancy [47, 48], until effective vaccines become available [49, 50].

In both healthcare and childcare workers, having one or more children residing at home doubled the risk of CMV seropositivity (Table 4). Therefore, in addition to exposure to children in the workplace, exposure to children at home may significantly contribute to overall risk. Certainly, CMV transmission occurs between occupationally exposed workers, their children, and children in childcare, and that, in any direction. A vicious cycle of viral transmission [51] can easily be envisioned; approximately, 50% of CMV-positive women transmit the virus through breast milk to their children [44, 52, 53] who may then reinfect the mother, a phenomenon described as “ping-pong” transmission [51]. The practice of proper infection prevention control measures, such as frequent hand washing, wearing protective gloves, avoiding kissing children on the mouth/cheeks, and not sharing utensils, foods, drinks, and washcloths, decreases the likelihood of CMV infection [50, 54–58]. Educating childcare workers to adhere to these simple preventive strategies at home and in the workplace should help reduce the transmission of CMV, the likelihood of primary and non-primary infection, and ultimately the risk of congenital CMV infection, as has been described for pregnant women and parents [54, 59, 60]. Consistent with earlier studies [1, 32, 61], we observed greater odds for CMV seropositivity among non-Caucasian ethnicities. Additionally, childcare workers who are married or in a common-law civil union had a significantly greater risk of CMV infection compared with people who are single, probably because couples are more likely to have children residing at home and attending daycare. Among healthcare workers, age greater than 30 years was associated with CMV seropositivity, agreeing with previous reports [1, 62].

The main strength of this meta-analysis is that it included studies in healthcare and childcare workers from all countries, without an inferior cutoff for year of publication. This contrasts with the work of Stark et al. [34] who reported on childcare workers only and included articles published since the year 2000. As shown in Fig. 1 (also supplemental Table S4), 70% of the studies (Fig. 1 & Additional file Table S4) included in this study were published prior to that year, making the present study more complete [19]. To our knowledge, this is the first comprehensive study to provide pooled estimates of CMV prevalence and incidence of primary infection and to compare the risk of CMV seropositivity and primary infection in healthcare workers versus controls.

The results presented here should be considered in the context of certain limitations. First, most of the studies included did not stratify data according to age, gender, or age of children in daycare, and we could therefore not specifically study women of reproductive age working at childcare and exposed to children younger than 3 years old (when risk of transmission is highest), nor provide data on the incidence of congenital infection. Second, we included articles in French and English only, and studies were predominantly North American and European, thus imposing some limit on generalizability. Third, subgroup analyses of pooled studies stratified by continent, diagnostic method, study design, and quality did not eliminate heterogeneity in our study outcomes. Heterogeneity observed across continents could be explained by local daycare policies that may differ regarding the number of children assigned per daycare worker and the amount of time spent with the children. Different study designs with differing methods of data collection, sampling, statistical analyses, and parameters assessed to determine the quality of the studies could also contribute to heterogeneity across studies. Other factors included the inability to fully distinguish the level of exposure to young children from exposure to other people at risk of CMV infection and the fact that no clear distinctions could be made between large childcare centers and home-based childcare centers. A major objective of meta-analysis was to identify and compare trends among studies rather than to synthesize data from studies to obtain a single conclusive estimate [63–66], and this has been achieved in our analyses. Lastly, although the methods used to diagnose CMV were reliable, each has its own limitations [67] such as how active vs. latent CMV infection is interpreted [67].

## Conclusions

This meta-analysis provides updated estimates of indicators of CMV infection in healthcare and childcare and workers. Prevalence and incidence of CMV infection was more common in childcare than in healthcare workers, which we believe is due to better adherence to infection prevention measures in the healthcare environment. Healthcare and childcare workers having one or more children living at home, being non-Caucasian, and being married or in a common-law relationship were positively associated CMV seropositivity. The relative contribution of these risk factors to the overall risk of CMV primary infection and congenital CMV infection among healthcare and childcare workers remains to be established. Our results suggest that attention to good hygienic measures can reduce the risk for CMV transmission in childcare.

## Supplementary Information

**Additional file 1: Supplementary Table 1.** PRISMA 2020 check lists. **Supplementary Table 2.** Search strategy. **Supplementary Table 3.** Results of quality assessment of the observational included studies. **Supplementary Table 4.** Characteristics of the selected articles. **Supplementary Table 5.** Funnel plot asymmetry test for publication bias. **Supplementary Table 6.** Meta-regression
