# Supplementary material for: Prevalence, incidence, and risk factors associated with cytomegalovirus infection in healthcare and childcare worker: a systematic review and meta-analysis
Source: Syst Rev. 2022 Jun 27;11:131. doi: 10.1186/s13643-022-02004-4 (PMC9235282; doi:10.1186/s13643-022-02004-4)
Supplement: Supplementary file 1 — Additional file 1: Supplementary Table 1. PRISMA 2020 check lists. Supplementary Table 2. Search strategy. Supplementary Table 3. Results of quality assessment of the observational included studies. Supplementary Table 4. Characteristics of the selected articles. Supplementary Table 5. Funnel plot asymmetry test for publication bias. Supplementary Table 6. Meta-regression [file 13643_2022_2004_MOESM1_ESM.docx]

Additional file

**Supplementary Table 1. PRISMA 2020 check lists**

| **Section and Topic** | **Item #** | **Checklist item** | **Location where item is reported** |
| --- | --- | --- | --- |
| **TITLE** | | |  |
| Title | 1 | Identify the report as a systematic review. | 1 |
| **ABSTRACT** | | |  |
| Abstract | 2 | See the PRISMA 2020 for Abstracts checklist. | 2 |
| **INTRODUCTION** | | |  |
| Rationale | 3 | Describe the rationale for the review in the context of existing knowledge. | 4 |
| Objectives | 4 | Provide an explicit statement of the objective(s) or question(s) the review addresses. | 4,5 |
| **METHODS** | | |  |
| Eligibility criteria | 5 | Specify the inclusion and exclusion criteria for the review and how studies were grouped for the syntheses. | 5 |
| Information sources | 6 | Specify all databases, registers, websites, organisations, reference lists and other sources searched or consulted to identify studies. Specify the date when each source was last searched or consulted. | 6 |
| Search strategy | 7 | Present the full search strategies for all databases, registers and websites, including any filters and limits used. | 6 |
| Selection process | 8 | Specify the methods used to decide whether a study met the inclusion criteria of the review, including how many reviewers screened each record and each report retrieved, whether they worked independently, and if applicable, details of automation tools used in the process. | 6 |
| Data collection process | 9 | Specify the methods used to collect data from reports, including how many reviewers collected data from each report, whether they worked independently, any processes for obtaining or confirming data from study investigators, and if applicable, details of automation tools used in the process. | 6,7 |
| Data items | 10a | List and define all outcomes for which data were sought. Specify whether all results that were compatible with each outcome domain in each study were sought (e.g. for all measures, time points, analyses), and if not, the methods used to decide which results to collect. | 6,7 |
|  | 10b | List and define all other variables for which data were sought (e.g. participant and intervention characteristics, funding sources). Describe any assumptions made about any missing or unclear information. | 6,7 |
| Study risk of bias assessment | 11 | Specify the methods used to assess risk of bias in the included studies, including details of the tool(s) used, how many reviewers assessed each study and whether they worked independently, and if applicable, details of automation tools used in the process. | 6,7 |
| Effect measures | 12 | Specify for each outcome the effect measure(s) (e.g. risk ratio, mean difference) used in the synthesis or presentation of results. | 7,8 |
| Synthesis methods | 13a | Describe the processes used to decide which studies were eligible for each synthesis (e.g. tabulating the study intervention characteristics and comparing against the planned groups for each synthesis (item #5)). | 8 |
|  | 13b | Describe any methods required to prepare the data for presentation or synthesis, such as handling of missing summary statistics, or data conversions. | 8 |
|  | 13c | Describe any methods used to tabulate or visually display results of individual studies and syntheses. | 7,8 |
|  | 13d | Describe any methods used to synthesize results and provide a rationale for the choice(s). If meta-analysis was performed, describe the model(s), method(s) to identify the presence and extent of statistical heterogeneity, and software package(s) used. | 7,8 |
|  | 13e | Describe any methods used to explore possible causes of heterogeneity among study results (e.g. subgroup analysis, meta-regression). | 7,8 |
|  | 13f | Describe any sensitivity analyses conducted to assess robustness of the synthesized results. | 7,8 |
| Reporting bias assessment | 14 | Describe any methods used to assess risk of bias due to missing results in a synthesis (arising from reporting biases). | 7,8 |
| Certainty assessment | 15 | Describe any methods used to assess certainty (or confidence) in the body of evidence for an outcome. |  |
| **RESULTS** | | |  |
| Study selection | 16a | Describe the results of the search and selection process, from the number of records identified in the search to the number of studies included in the review, ideally using a flow diagram. | 9 |
|  | 16b | Cite studies that might appear to meet the inclusion criteria, but which were excluded, and explain why they were excluded. | 9 |
| Study characteristics | 17 | Cite each included study and present its characteristics. | 9 |
| Risk of bias in studies | 18 | Present assessments of risk of bias for each included study. | 9 |
| Results of individual studies | 19 | For all outcomes, present, for each study: (a) summary statistics for each group (where appropriate) and (b) an effect estimate and its precision (e.g. confidence/credible interval), ideally using structured tables or plots. | 9,10,11 |
| Results of syntheses | 20a | For each synthesis, briefly summarise the characteristics and risk of bias among contributing studies. |  |
|  | 20b | Present results of all statistical syntheses conducted. If meta-analysis was done, present for each the summary estimate and its precision (e.g. confidence/credible interval) and measures of statistical heterogeneity. If comparing groups, describe the direction of the effect. | 9,10,11 |
|  | 20c | Present results of all investigations of possible causes of heterogeneity among study results. | 9,10,11, 12 |
|  | 20d | Present results of all sensitivity analyses conducted to assess the robustness of the synthesized results. | 11,12 |
| Reporting biases | 21 | Present assessments of risk of bias due to missing results (arising from reporting biases) for each synthesis assessed. |  |
| Certainty of evidence | 22 | Present assessments of certainty (or confidence) in the body of evidence for each outcome assessed. |  |
| **DISCUSSION** | | |  |
| Discussion | 23a | Provide a general interpretation of the results in the context of other evidence. | 10,11 |
|  | 23b | Discuss any limitations of the evidence included in the review. | 12 |
|  | 23c | Discuss any limitations of the review processes used. | 12 |
|  | 23d | Discuss implications of the results for practice, policy, and future research. | 10,11,13 |
| **OTHER INFORMATION** | | |  |
| Registration and protocol | 24a | Provide registration information for the review, including register name and registration number, or state that the review was not registered. | 13,14,15 |
|  | 24b | Indicate where the review protocol can be accessed, or state that a protocol was not prepared. | 16 |
|  | 24c | Describe and explain any amendments to information provided at registration or in the protocol. | 16 |
| Support | 25 | Describe sources of financial or non-financial support for the review, and the role of the funders or sponsors in the review. | 3,17 |
| Competing interests | 26 | Declare any competing interests of review authors. | 17 |
| Availability of data, code and other materials | 27 | Report which of the following are publicly available and where they can be found: template data collection forms; data extracted from included studies; data used for all analyses; analytic code; any other materials used in the review. | Available upon request |

**Supplementary Table 2. Search strategy**

2.1 PubMed March 7, 2022

| 1 | CMV | Cytomegalovirus[Mh] OR Cytomegalovirus Infections[Mh] OR Cytomegal*[tiab] OR Salivary Gland Virus*[tiab] OR "HHV 5"[tiab] OR HHV5[tiab] OR Human Herpesvirus5[tiab] OR Human Herpesvirus 5[tiab] OR Human Herpes virus 5[tiab] OR CMV[tiab] OR cCMV[tiab] OR Inclusion Disease*[tiab] OR Cytomegal*[OT] OR "Salivary Gland Virus"[OT] OR "Salivary Gland Viruses"[OT] OR "HHV 5"[OT] OR HHV5[OT] OR Human Herpesvirus5[OT] OR Human Herpesvirus 5[OT] OR "Human Herpes virus 5"[OT] OR CMV[OT] OR cCMV[OT] OR Inclusion Disease*[OT] |
| --- | --- | --- |
| 2 | Occupational exposure | Occupational Groups[Mh] OR "Non-Medical Public and Private Facilities"[Mesh] OR Occupational Exposure[Mh:noexp] OR Occupational Diseases[mh:noexp] OR Occupational Health[Mh] OR Employment[Mh:noexp] OR Workplace[Mh] OR Women, Working[Mh] OR Employee*[tiab] OR Employment*[tiab] OR Working[tiab] OR Worker*[tiab] OR Workplace*[tiab] OR Work place*[tiab] OR Personnel[tiab] OR Provider*[tiab] OR Occupation*[tiab] OR Staff*[tiab] OR Childcare[tiab] OR Day care[tiab] OR Kindergarten*[tiab] OR Preschool*[tiab] OR Pre-school*[tiab] OR early school*[tiab] OR Employee*[OT] OR Employment*[OT] OR Working[OT] OR Worker*[OT] OR Workplace*[OT] OR Work place*[OT] OR Personnel[OT] OR Provider*[OT] OR Occupation*[OT] OR Staff*[OT] OR Childcare[OT] OR Day care[OT] OR Kindergarten*[OT] OR Preschool[OT] OR Pre-school[OT] OR early school[OT] |
| 3 | Animals | (Animals[mh] NOT humans[mh]) OR rat[tiab] OR rats[tiab] OR mouse[tiab] OR mice[tiab] OR murine[tiab] OR rat[OT] OR rats[OT] OR mouse[OT] OR mice[OT] OR murine[OT] |
| 4 | Combinaison and limitations | (((#1 AND #2) NOT #3) AND (english[LA] OR french[LA]))  782 results |

2.2 Medline (OVID) March 7, 2022

| 1 | CMV | Exp Cytomegalovirus/ OR Exp Cytomegalovirus Infections/ OR (Cytomegal* OR Salivary Gland Virus* OR "HHV 5" OR HHV5 OR Human Herpesvirus5 OR Human Herpesvirus 5 OR Human Herpes virus 5 OR CMV OR cCMV OR Inclusion Disease*).ti,ab,kw,kf |
| --- | --- | --- |
| 2 | Occupational exposure | Exp Occupational Groups/ OR exp "non-medical public and private facilities"/ OR Occupational Exposure/ OR Occupational Diseases/ OR Occupational Health/ OR Employment/ OR Workplace/ OR Women, Working/ OR (Employee* OR Employment* OR Working OR Worker* OR Workplace* OR Work place* OR Personnel OR Provider* OR Occupation* OR Staff* OR Childcare OR Day care OR Kindergarten* OR Preschool* OR Pre-school* OR early school*).ti,ab,kw,kf |
| 3 | Animals | (Exp Animals/ NOT Exp humans/) OR (rat OR rats OR mouse OR mice OR murine).ti,ab,kw,kf |
| 4 | Combination and limitations | (((1 AND 2) NOT 3) AND (english OR french).lg) 783 results |

2.3 All EBM Reviews March 7, 2022

| 1 | CMV | Exp Cytomegalovirus/ OR Exp Cytomegalovirus Infections/ OR (Cytomegal* OR Salivary Gland Virus* OR "HHV 5" OR HHV5 OR Human Herpesvirus5 OR Human Herpesvirus 5 OR Human Herpes virus 5 OR CMV OR cCMV OR Inclusion Disease*).ti,ab,kw,kf |
| --- | --- | --- |
| 2 | Occupational exposure | Exp Occupational Groups/ OR exp "non-medical public and private facilities"/ OR Occupational Exposure/ OR Occupational Diseases/ OR Occupational Health/ OR Employment/ OR Workplace/ OR Women, Working/ OR (Employee* OR Employment* OR Working OR Worker* OR Workplace* OR Work place* OR Personnel OR Provider* OR Occupation* OR Staff* OR Childcare OR Day care OR Kindergarten* OR Preschool* OR Pre-school* OR early school*).ti,ab,kw,kf |
| 3 | Animals | (Exp Animals/ NOT Exp humans/) OR (rat OR rats OR mouse OR mice OR murine).ti,ab,kw,kf |
| 4 | Combinaison and limitations | (((1 AND 2) NOT 3) AND (english OR french).lg) 59 results |

2.4 Embase March 7, 2022

| 1 | CMV | Exp Cytomegalovirus/ OR Exp Cytomegalovirus Infection/ OR (Cytomegal* OR Salivary Gland Virus* OR "HHV 5" OR HHV5 OR Human Herpesvirus5 OR Human Herpesvirus 5 OR Human Herpes virus 5 OR CMV OR cCMV OR Inclusion Disease*).ti,ab,kw |
| --- | --- | --- |
| 2 | Occupational exposure | Exp named groups by occupation/ OR day care/ OR Occupational Exposure/ OR Occupational Disease/ OR Occupational Health/ OR Employment/ OR Workplace/ OR female worker/ OR (Employee* OR Employment* OR Working OR Worker* OR Workplace* OR Work place* OR Personnel OR Provider* OR Occupation* OR Staff* OR Childcare OR Day care OR Kindergarten* OR Preschool* OR Pre-school* OR early school*).ti,ab,kw |
| 3 | Animals | (Exp Animal/ NOT Exp human/) OR (rat OR rats OR mouse OR mice OR murine).ti,ab,kw |
| 4 | Combinaison and limitations | (((1 AND 2) NOT 3) AND (english OR french).lg) 3358 results |

2.5 Web of Science March 7, 2022

| 1 | CMV | TS=(Cytomegal* OR Salivary Gland Virus* OR "HHV 5" OR HHV5 OR "Human Herpesvirus5" OR "Human Herpesvirus 5" OR "Human Herpes virus 5" OR CMV OR cCMV OR "Inclusion Disease" OR "Inclusion Diseases") |
| --- | --- | --- |
| 2 | Occupational exposure | TS=("Employee" OR"Employees" OR "Employment" OR "Working" OR "Worker" OR "Workers" OR Workplace* OR "work place" or "work places" OR Personnel OR "Provider"OR "Providers" OR Occupation* OR Staff* OR Childcare OR "Day care" OR Kindergarten* OR Preschool* OR Pre-school* OR "early school" OR "early schools") |
| 3 | Animals | TS=(rat OR rats OR mouse OR mice OR murine) |
| 4 | Combinaison and limitations | (((1 AND 2) NOT 3) AND (english OR french).lg) 925 results |

2.6 CINAHL COMPLETE March 7, 2022

| S1 | CMV | MH(Cytomegaloviruses) OR MH(Cytomegalovirus Infections+) OR TI(Cytomegal* OR Salivary Gland Virus* OR "HHV 5" OR HHV5 OR Human Herpesvirus5 OR Human Herpesvirus 5 OR Human Herpes virus 5 OR CMV OR cCMV OR Inclusion Disease*) OR AB(Cytomegal* OR Salivary Gland Virus* OR "HHV 5" OR HHV5 OR Human Herpesvirus5 OR Human Herpesvirus 5 OR Human Herpes virus 5 OR CMV OR cCMV OR Inclusion Disease*) |
| --- | --- | --- |
| S2 | Occupational exposure | MH(Named Groups by Occupation+) OR MH(Child Day Care) OR MH(Occupational Exposure) OR MH(Occupational Diseases) OR MH(Occupational Health+) OR MH(Employment) OR MH(Work Environment+) OR MH(Women, Working+) OR TI(Employee* OR Employment* OR Working OR Worker* OR Workplace* OR Work place* OR Personnel OR Provider* OR Occupation* OR Staff* OR Childcare OR Day care OR Kindergarten* OR Preschool* OR Pre-school* OR early school*) OR AB(Employee* OR Employment* OR Working OR Worker* OR Workplace* OR Work place* OR Personnel OR Provider* OR Occupation* OR Staff* OR Childcare OR Day care OR Kindergarten* OR Preschool* OR Pre-school* OR early school*) |
| S3 | Animals | (MH(Vertebrates+) NOT MH(human)) OR TI(rat OR rats OR mouse OR mice OR murine) OR AB(rat OR rats OR mouse OR mice OR murine) |
| S4 | Combinaison and limitations | ((S1 AND S2) NOT S3) Opérateurs de restriction - Langue: English, French 226 results |

**Supplementary Table 3. Results of quality assessment of the observational included studies**

|  | **Year** | **Author** | **CK1** | **CK2** | **CK3** | **CK4** | **CK5** | **CK6** | **CK7** | **CK8** | **CK9** | **CK10** | **CK11** | **CK12** | **CK13** | **CK14** | **Quality** |
| --- | --- | --- | --- | --- | --- | --- | --- | --- | --- | --- | --- | --- | --- | --- | --- | --- | --- |
| 1. | 1984 | Adler | N | N | NR | N | N | NR | NR | NA | NR | NA | NR | NR | NR | NR | Poor |
| 2. | 1989 | Adler | N | Y | NR | Y | NR | NR | NR | NR | NR | NR | NR | NR | NR | NR | Poor |
| 3. | 2000 | Adler | Y | Y | Y | Y | NR | Y | Y | N | Y | Y | Y | NR | Y | N | Fair |
| 4. | 1981 | Ahlfors, K. et al. | Y | Y | NR | Y | N | Y | Y | Y | Y | Y | Y | NR | N | Y | Good |
| 5. | 1990 | Balcarek et l. | Y | Y | NR | Y | N | Y | Y | Y | Y | N | Y | Y | Y | Y | Good |
| 6. | 1999 | Bale et al. | Y | Y | NR | Y | NR | Y | Y | Y | Y | NR | Y | Y | N | N | Good |
| 7. | 1986 | Balfour et al. | Y | Y | Y | N | N | Y | Y | Y | Y | N | Y | NR | NR | Y | Good |
| 8. | 1987 | Blackman et al | Y | Y | NR | Y | N | Y | Y | NR | Y | NR | Y | NR | N | N | Fair |
| 9. | 1987 | Brady et al. | Y | Y | NR | Y | N | Y | Y | NR | Y | NR | Y | NR | N | NR | Fair |
| 10. | 1985 | C. Hutto et al. | N | N | NR | N | N | NR | NR | NR | NR | NR | NR | NR | NR | NR | Poor |
| 11. | 1999 | De Schryver, A., et al. | N | Y | NR | N | N | Y | Y | NR | Y | N | Y | NR | NR | Y | Fair |
| 12. | 2011 | de Villemeur, et al. | Y | Y | Y | Y | N | Y | Y | Y | Y | N | Y | NR | NR | Y | Good |
| 13. | 1983 | Dworsky, et al. | N | Y | NR | N | N | Y | Y | N | Y | N | Y | NR | N | Y | Fair |
| 14. | 1988 | Embil et al. | Y | Y | NR | Y | N | NR | NR | NR | Y | NR | Y | NR | N | NR | Fair |
| 15. | 1996 | Ford-Jones ET AL. | Y | Y | NR | Y | N | Y | Y | N | Y | NR | Y | N | NR | Y | Good |
| 16. | 1984 | Friedman et al. | N | Y | Y | Y | N | Y | Y | NR | Y | NR | Y | NR | NR | N | Fair |
| 17. | 1994 | Gerberding, J. L. | Y | Y | NR | Y | N | Y | Y | Y | NR | NR | Y | NR | N | NR | Good |
| 18. | 1985 | Hatherley, L. I. | N | Y | N | Y | N | Y | NR | NR | Y | NR | Y | NR | NR | N | Fair |
| 19. | 1986 | Hatherley, L. I. | Y | Y | NR | Y | N | NR | Y | NR | NR | NR | Y | NR | NR | NR | Poor |
| 20. | 1996 | Jackson, L. A., et al. | Y | Y | N | Y | Y | Y | N | N | Y | N | Y | NR | NR | N | Fair |
| 21. | 1985 | Jones et al. | N | N | N | Y | N | NR | NR | NR | N | NR | N | NR | N | NR | Poor |
| 22. | 2005 | Joseph, S.A. et al. | Y | Y | N | Y | N | Y | NR | Y | Y | N | Y | NR | NR | Y | Good |
| 23. | 2002 | Kiss P. et al. | Y | Y | N | Y | N | NR | NR | Y | Y | N | Y | NR | NR | Y | Good |
| 24. | 2016 | Lamarre V. et al. | Y | Y | Y | Y | N | Y | Y | Y | Y | N | Y | NR | NR | Y | Good |
| 25. | 2011 | Lepage N. et al. | Y | Y | NR | Y | N | NR | NR | Y | Y | N | Y | NR | NR | Y | Fair |
| 26. | 2002 | Leroux, M. C., et al. | Y | N | NR | NR | NR | NR | NR | NR | NR | N | NR | NR | NR | NR | Poor |
| 27. | 1984 | Lipscomb, J. A., et al. | N | N | Y | Y | N | NR | NR | NR | NR | NR | Y | NR | NR | Y | Poor |
| 28. | 2003 | Morgan, M. A.,et al. | Y | Y | NR | NR | NR | NR | NR | NR | NR | NR | Y | NR | NR | NR | Poor |
| 29. | 1986 | Murph, J. R., et al. | N | N | NR | Y | N | NR | Y | N | N | N | Y | NR | NR | N | Poor |
| 30. | 1985 | Murph, J. R et al. | N | N | N | NR | N | NR | N | N | N | N | NR | N | N | N | Poor |
| 31. | 1991 | Murph, J. R., et al. | Y | Y | NR | Y | N | Y | Y | N | NR | N | Y | NR | NR | NR | Fair |
| 32. | 1987 | Nelson, D. B et al. | N | Y | NR | NR | N | Y | NR | N | N | N | Y | NR | N | Y | Fair |
| 33. | 2009 | Okwori et al. | N | N | NR | Y | N | NR | NR | NR | NR | NR | Y | NR | NR | N | Poor |
| 34. | 1990 | Pass, R. F., et al. | Y | Y | NR | Y | N | Y | Y | NR | Y | NR | Y | NR | NR | Y | Good |
| 35. | 2000 | Sobaszek, A., et al. | Y | Y | Y | Y | N | Y | NR | Y | Y | NR | Y | NR | NR | N | Good |
| 36. | 1994 | Soto, J. C., et al. | N | N | N | Y | N | Y | NR | NR | NR | N | NR | NR | NR | N | Poor |
| 37. | 2006 | Stelma, F. F., et al. | Y | Y | NR | Y | N | NR | NR | Y | Y | N | Y | NR | NR | N | Fair |
| 38. | 2016 | Stranzinger, J., Kindel, J., | Y | Y | NR | Y | N | Y | NR | Y | Y | N | Y | NR | N | Y | Good |
| 39. | 2016 | Stranzinger, J., Kozak, A., | Y | Y | NR | NR | N | N | N | Y | Y | N | Y | NR | N | Y | Fair |
| 40. | 2020 | Takao, M., et al. | Y | Y | NR | Y | N | Y | Y | Y | Y | Y | Y | NR | N | NR | Good |
| 41. | 1978 | Tolkoff-Rubin, et al. | N | N | NR | Y | N | NR | NR | NR | Y | NR | Y | NR | NR | N | Poor |
| 42. | 2010 | Uslu, H., et al. | Y | Y | NR | N | N | NR | NR | NR | Y | N | Y | NR | NR | N | Poor |
| 43. | 2012 | Van Rijckevorsel, G. et al. | Y | Y | Y | Y | N | NR | NR | Y | Y | NR | Y | NR | NR | Y | Good |
| 44. | 1998 | Volpi, A., et al. | Y | Y | N | Y | NR | NR | NR | Y | Y | N | Y | NR | NR | N | Fair |
| 45. | 2010 | Wicker, S. | N | N | N | Y | N | NR | NR | N | N | NR | N | NR | N | N | Poor |
| 46 | 1975 | Yeager, A. S. | NR | Y | N | Y | N | Y | Y | Y | Y | N | Y | NR | NR | Y | Good |
| 47 | 2020 | Zając, P. W., et al. | Y | Y | NR | Y | N | Y | Y | Y | Y | N | Y | NR | NR | N | Good |
| 48 | 2021 | Zenebe, M. H., et al. | Y | Y | Y | Y | Y | Y | NR | NR | Y | NR | Y | NR | Y | Y | Good |

Quality of included studies was assessed using the National Institutes of Health (NIH) Quality Assessment tool for Observational Cohort and Cross-Sectional Studies (https://[www.nhlbi.nih.gov/health-pro/guidelines/in-develop/cardiovascular-risk-reduction/tools/cohort).](http://www.nhlbi.nih.gov/health-pro/guidelines/in-develop/cardiovascular-risk-reduction/tools/cohort)) CK 1. Was the research question or objective in this paper clearly stated? CK 2. Was the study population clearly specified and defined? CK 3. Was the participation rate of eligible persons at least 50%? CK 4. Were all the subjects selected or recruited from the same or similar populations (including the same time period)? Were inclusion and exclusion criteria for being in the study prespecified and applied uniformly to all participants? CK 5. Was a sample size justification, power description, or variance and effect estimates provided? CK 6. For the analyses in this paper, were the exposure(s) of interest measured prior to the outcome(s) being measured? CK 7. Was the timeframe sufficient so that one could reasonably expect to see an association between exposure and outcome if it existed? CK 8. For exposures that can vary in amount or level, did the study examine different levels of the exposure as related to the outcome (e.g., categories of exposure, or exposure measured as continuous variable)? CK 9. Were the exposure measures (independent variables) clearly defined, valid, reliable, and implemented consistently across all study participants? CK 10. Was the exposure(s) assessed more than once over time? CK 11. Were the outcome measures (dependent variables) clearly defined, valid, reliable, and implemented consistently across all study participants? CK 12. Were the outcome assessors blinded to the exposure status of participants? CK 13. Was loss to follow-up after baseline 20% or less? CK 14. Were key potential confounding variables measured and adjusted statistically for their impact on the relationship between exposure(s) and outcome(s)? CK, check list; CD, cannot be determined; NA, not applicable; NR, not reported; N, no; Y, yes.

**Supplementary Table 4. Characteristics of the selected articles**

| id | **Author year** | **study year data collection** | **Year of publication** | **country** | **Study design** | **Place of the study** | **No of exposed** | **No of unexposed** | **Exposure: duration of employment, job duties** | **outcome measurement** | **Quality**  **: Good**  **: Fair**    **: Poor** |
| --- | --- | --- | --- | --- | --- | --- | --- | --- | --- | --- | --- |
| 1 | Adler, S. P. (1984).(1) |  | 1985 | USA | Cross-sectional | Hospital (n=1) | Nurses  n=72 | ─ | ─ | ─ |  |
| 2 | Adler, S. P. (1988).(2) | 1986 | 1988 | USA | Cohort | Childcare (n=1) | Daycare workers n=12 | ─ | Duration of employment: 2 years as average, range 3 months to 4 years | Latex agglutination |  |
| 3 | Adler, S. P. (1989)(3) | 1986-1988 | 1989 | USA | Cohort | Childcare (n=34) & Hospital | Childcare workers n=610 | Women employed by the Medical college of Virginia hospitals  n=565 | Duration of employment: < 1 years=6.2%, 1 to 3 years=23%, >3 to 5 years=4.5%, >5 years=7.9% | ELISA |  |
| 4 | Ahlfors, K., Ivarsson, S. A., Johnsson, T., & Renmarker, K. (1981).(4) | 1978-1980 | 1981 | Sweden | Cohort | Hospital (n=1) | Nurses  n=192 | Women who had no professional contact with infants  n=163 | ─ | Complement fixation tests |  |
| 5 | Balcarek, K. B., Bagley, R., Cloud, G., & Pass, R. F. (1990)(5) | Dec 1984 -Aug1987 | 1990 | USA | Cohort | Hospital (n=1) | Employee with patient care (nurses, physicians, clinical aides or assistants,)  n=669 | ─ | questionnaire self administrated, | ELISA (Cytomegelisa CMV Stat, Whittaker MA Bioproducts, |  |
| 6 | Bale, J. F., Jr., Zimmerman, B., Dawson, J. D., Souza, I. E., Petheram, S. J., & Murph, J. R. (1999).(6) | March 1991- August 1994 | 1999 | USA | Cohort | Child care homes (n=25) | Child care providers  n=132 | ─ | questionnaire self administrated,  Duration of employment-median and interquartile in months: 28.5(10-96.5) for seropositive providers vs 21.5 (7.5-52) for seronegative providers | Latex agglutination |  |
| 7 | Balfour, C. L., & Balfour, H. H., Jr. (1986).(7) | September 1979-August 1984 | 1986 | USA | Cohort | Hospital and university | Renal transplant/hemodialysis nurses  n=263  Neonatal intensive care nurses  n=204  Student nurses  n=225 | ─ | ─ | Complement Fixing and Indirect Immunofluorescent tests) |  |
| 8 | Blackman, J. A., Murph, J. R., & Bale, J. F., Jr. (1987).(8) |  | 1987 | USA | Cohort | Hospital (n=1) | Staff with direct occupational contact with disabled children (teachers, physical therapists, classroom aids, psychologists, physicians and nurses)  N=99 | Staff without occupational child contact (secretaries, librarians, janitors and kitchen workers)  n=77 | ─ | Latex agglutination |  |
| 9 | Brady, M. T., Demmler, G. J., & Anderson, D. C. (1987). (9) | July 1981 - July 1984 | 1987 | USA | Cohort | Hospital (n=1) | Nurse  n=152 | ─ | ─ | Anticomplement immunofluorescence method) |  |
| 10 | **C Hutto, MD, RE Ricks, RF Pass, 1985(10)** | 1984 | 1985 | USA | Cross-sectional | Childcare(n=5) | Childcare workers n=82 | ─ | ─ | ELISA |  |
| 11 | De Schryver, A., Glazemakers, J., De Bacquer, D., De Backer, G., & Lust, E. (1999).(11) | From oct 1993 and may 1994 - 1995 | 1998 | Belgium | Cross-sectional | Institution for menatlly disabled children, and Home for eldery people (n=1) | Educators for mentally disabled children  n=283 | Nurses of home for eldery people  N=294 | ─ | ELISA |  |
| 12 | de Villemeur, A. B., Gratacap-Cavallier, B., Casey, R., Baccard-Longere, M., Goirand, L., Seigneurin, J. M., & Morand, P. (2011). (12) | 2005 - 2007 | 2011 | France | Cross-sectional | Drop-in child-care facilities (n=1) | Child-care staff n=395 | Employees of STMicroelectronics (STM) or the French Atomic Energy Commission  n=382 | ─ | Enzygnost anti-CMV/IgG (Dade-Behring) assays. |  |
| 13 | Dworsky, M. E., Welch, K., Cassady, G., & Stagno, S. (1983).(13) | 1982 | 1983 | USA | Cross-sectional | Hospital (n=1) | Nurses, medical student, community pediatricians, CMV research physician  n= l 452 | Middle-class pregnant women from a large private obstetrical practice  n=3733  Middle-class women who become pregnant a second tim  n=720 | ─ | Anticomplement immunofluorescence method |  |
| 14 | Embil, J. A., Pereira, L. H., Manley, K., & MacNeil, J. P. (1988).(14) | September 1985 - June 1986 | 1988 | Canada | Cross-sectional | Hospital (n=1) | Nurses  n=102 | ─ | ─ | Latex agglutination |  |
| 15 | Ford-Jones, E. L., Kitai, I., Davis, L., Corey, M., Farrell, H., Petric, M., . . . Gold, R. (1996)(15) |  | 1996 | Canada | Cross-sectional | Daycare centres (n=38) | Day-care providers n=205 | ─ | Contact with children for at least 10 h/week | Latex agglutination |  |
| 16 | Friedman, H. M., Lewis, M. R., Nemerofsky, D. M., & Plotkin, S. A. (1984)(16). | June 1979- January 1981 | 1984 | USA | Cross-sectional | Hospital (n=1) | Health care staff with close patient contact (nurse, blood drawing or iv insertion team  n=173 | Employees no intimate patient contact (secretaries, administrators, social workers)  n=35 | ─ | Complement fixation |  |
| 17 | Gerberding, J. L. (1994)(17) | May 1984 and September 1992 | 1994 | USA | Cohort | Hospital (n=1) | Health care providers: Physician n=369  Nurses n=256; Dentist n=155 | ─ | ─ | Anticomplement immunofluorescence assay |  |
| 18 | Hatherley, L. I. (1985).(18) | 1983 | 1985 | Australia | Coss-sectional | Hospital (n=1) | Midwives, special care nursery staff and mothercraft nurses n=290 | ─ | ─ | Complement fixation |  |
| 19 | Hatherley, L. I. (1986).(19) | 1981-1985 | 1986 | Australia | Cohort | Hospital (n=1) | Healthcare workers from antenatal and postnatal wards  n=154 | Non-nursing duties staff  n=12 | ─ | ELISA |  |
| 20 | Jackson, L. A., Stewart, L. K., Solomon, S. L., Boase, J., Alexander, E. R., Heath, J. L., . . . Shapiro, C. N. (1996).(20) | May-August 1994 | 1996 | USA | Cross-sectional | Child care facilities (n=49) | Childcare workers (n=360). | ─ | Duration of employment:  -1 year(15%)  -2-4 years(33%)  -5-9years (25%)  ->10 years(26%) | ELISA |  |
| 21 | Jones, L. A., Duke-Duncan, P. M., & Yeager, A. S. (1985). (21) |  | 1985 | USA | Cohort | Day care centers(n=6) and Infant development centers(n=10) | Day care workers n=58 | ─ | ─ | Indirect hemagglunation (IHA) |  |
| 22 | Joseph, S. A., Beliveau, C., Muecke, C. J., Rahme, E., Soto, J. C., Flowerdew, G., . . . Gyorkos, T. W. (2005). (22) | October through December 2001 | 2005 | Canada | Cross-sectional | Day care centers (n=81) | Daycare workers n=473 | ─ | Average child-to-educator ratios for children ,18 months old, 18–35 months old and 36 months old were 3.2, 6.1 and 7.5, respectively | ELISA |  |
| 23 | Kiss, P., De Bacquer, D., Sergooris, L., De Meester, M., & VanHoorne, M. (2002).(23) | 1996-1997 | 2002 | Belgium | Cross-sectional | Kindergarten (n=50) | Kindergarten teachers  n=211 | Administrative workers  n=283 | Contact with children <6 years old: 34.3% for kindergarten teachears and 27% administrative workers | Enzyme-linked fluorescent assay (ELFA) |  |
| 24 | Lamarre, V., et al. (2016).(24) | May 2010 - February 2013 | 2016 | Canada | Cohort Study | Day care & hospital | Childcare workers  n=23  Nurse or midwife n=166 | ─ | ─ | ELISA- |  |
| 25 | Lepage, N., Leroyer, A., Cherot-Kornobis, N., Lartigau, I., Miczek, S., & Sobaszek, A. (2011)(25) | 1 January to 31 December 2003 | 2011 | France | Cross-sectional | Hospital (n=1) | Health care staff n=386 | Women carrying out clerical or administrative work at the Hospital  n=164 | Duration of employment (years, mean ± s.d.):  - exposed participants 14.8±10.2  - unexposed participants10.7±9.5 | ELISA |  |
| 26 | Leroux, M. C., Reinert, P., Boucher, J., & Lasry, S. (2002)(26) |  | 2002 | France | Cross-sectional | Childcare(n=22) | Childcare workers (n=17130) | Blood Donors | _ | Immunofluorescence des antigènes précoces |  |
| 27 | Lipscomb, J. A., Linnemann, C. C., Jr., Hurst, P. F., Myers, M. G., Stringer, W., Moore, P., & Hammond, J. (1984).(27) |  | 1984 | USA | cross-sectional | Hospital (n=1) | Nurses=288 | ─ | Duration of employment (years):  -0-0.5 =167  -0.5-3 =87  -3-5 =36  -5-10 =38  ->10 =33 | Indirect immunofluorescent assay (IFA) |  |
| 28 | Morgan, M. A.,et al. (2003). (28) |  | 2003 | Egypt?? | Cross-sectional | Hospital (n=1) | Healthcare workers n=19 | ─ | ─ | ELISA and DNA by PCR | _ |
| 29 | Murph, J. R., Bale, J. F., Jr., Murray, J. C., Stinski, M. F., & Perlman, S. (1986). (29) | 1984/1985 | 1986 | USA | Cross-sectional | Childcare (n=1) | Day care workers n=5 | ─ | Child/caretaker ratios:  -4:1 for children < 2 years old  -6:1 for children 2-3 years old  -8:1 for children 3-4 years old  -12:1 for children >4 years | Latex agglutination test |  |
| 30 | Murph, J. R., Bale, J. F., Perlman, S., & Swack, N. S. (1985)(30) |  | 1985 | USA | Cross-sectional | Childcare (n=1) | Daycare workers n=5 | ─ | ─ | Complement Fixation |  |
| 31 | Murph, J. R., Baron, J. C., Brown, C. K., Ebelhack, C. L., & Bale, J. F., Jr. (1991). (31) |  | 1991 | USA | Cohort | Childcare  (n=6) | Daycare workers n=252 | ─ | ─ | Latex agglutination |  |
| 32 | Nelson, D. B., Peckham, C. S., Pearl, K. N., Chin, K. S., Garrett, A. J., & Warren, D. E. (1987).(32) |  | 1987 | UK | Cross-sectional | Day care nurseries  (n=5) | Teachers  n=41 | Pregnant women in their first pregnancy, matched for race and social class, attending a maternity unit in the same area.  n=500 | _ | Restriction endonuclease |  |
| 33 | Okwori,et al (2009).**(33)** | November, 2004 and January, 2005 | 2009 | Nigeria | Cross-sectional | Hospital (n=1) | Healthcare workers  n=4 |  | ─ | ELISA |  |
| 34 | Pass, R. F., Hutto, C., Lyon, M. D., & Cloud, G. (1990)(34) | September 1980 - april 1988 | 1990 | USA | Cohort | Childcare (n=32) | Daycare workers  n=509 | ─ | Average duration of employment:  - 2.1 years among seropositive workers  - 0.45 yearsamong seronegative | ELISA & anticomplement immunofluorescence technique |  |
| 35 | Sobaszek, A., Fantoni-Quinton, S., Frimat, P., Leroyer, A., Laynat, A., & Edme, J. L. (2000).(35) | May 1997 and May 1998 | 2000 | France | Cross-sectional | Hospital (n=1) | Healthcare workers  n=171 | Personnel of technical task n=229 |  | ELISA |  |
| 36 | Soto, J. C., Delage, G., Vincelette, J., & Belanger, L. (1994).(36) | 1989-1991 | 1994 | Canada | Cross-sectional | Childcare (n=29) | Daycare workers n=148 | ─ |  | CMV antibody test, no specify |  |
| 37 | Stelma, F. F., Smismans, A., Goossens, V. J., Bruggeman, C. A., & Hoebe, C. J. (2009).(37) | October 2000 and April 2003 | 2009 | Netherland | Cross-sectional | Childcare (n=66) & Nursing school (n=1) | Female childcare workers  n=310 | ─ | Duration of employment: 6.4 years (SD 5.2 years) and 24 h per week (SD 9.3 days) | Automated microparticle enzymatic immunoassay AxSym CMV IgG version (Abbott laboratories, Abbott Park, IL, USA) |  |
| 38 | Stranzinger, J., Kindel, J., Henning, M., Wendeler, D., & Nienhaus, A. (2016).(38) | 2014 | 2016 | Germany | Cross-sectional | Hospital (n=1) | Nurses n=91 Doctor n=50  School student apprentice n=40  Other n=14 | ─ | ─ | ELISA |  |
| 39 | Stranzinger, J., Kozak, A., Schilgen, B., Paris, D., Niessen, T., Schmidt, L., . . . Nienhaus, A. (2016)(39) | 2010-2013 | 2016 | Germany | Cross-sectional | Hospital (n=1) | Pregnant childcare workers  n=509 | Female first-time blood donors n=14,358 | ─ | ELISA |  |
| 40 | Takao, M., Yoshioka, N., Hagiya, H., Deguchi, M., Kagita, M., Tsukamoto, H., . . . Chemotherapy. (2020)(40) | 2013-2018 | 2020 | Japan | Cohort | Hospital | Healthcare workers n=1153 | ─ | ─ | Alinity CMV-G  (Abbott Diagnostic Division, Tokyo, Japan) |  |
| 41 | Tolkoff-Rubin, N. A., Rubin, R. H., Keller, E. E., Baker, G. P., Stewart, J. A., & Hirsch, M. S. (1978). (41) | March 1, 1975 to February 28, 1976 | 1978 | USA | Cohort | Hospital (acute hemodialysis unit) (n=1) | Dialyse nurse n=6, Transplant nurse n=6  Staff physician n=3  Fellow=8  Ancillary personnel n=3 | ─ | ─ | Complement fixation |  |
| 42 | Uslu, H., Uyanik Hamidullah, M., Akpinar, R. B., & Celebioglu, A. (2010).(42) |  | 2010 | Turquie | Cross-sectional | School of Nursing (n=1) | Nursing student  n=89 | ─ | ─ | Enzyme Linked Fluorescent Assay (ELFA) VIDAS® system (bioMérieux). |  |
| 43 | Van Rijckevorsel, G. G., L. P. Bovee, M. Damen, G. J. Sonder, M. F. Schim van der Loeff and A. van den Hoek (2012)(43) | 2007 | 2012 | Netherland | cross-sectional | Day care  (n=38) | Women childcare workers  n=212 | Women not working in daycare came from a cross-sectional survey of the adult Amsterdam general population, the Amsterdam Health Monitor (AHM)  n=288 | ─ | ELISA |  |
| 44 | Volpi, A., Pica, F., Cauletti, M., Pana, A., & Rocchi, G. (1988). (44) |  | 1988 | Italia | Cross-sectional | Day care  (n=9) | Childcare workers n=82  Staff in training n=229 | Housewife matched by age and parity n=82 | Duration of employment: 7.2 years (range: 1-15 years) | Anticomplement-immunofluorescence |  |
| 45 | Wicker, S. (2012).(45) | March 2007 and July 2011 | 2012 | Germany | Cross-sectional | Hospital (n=1) | Healthcare workers  n=424 | ─ | ─ | ─ |  |
| 46 | Yeager, A. S. (1975).(46) | July 1969 and March 1974 | 1975 | USA | Cohort | Hospital (n=1) | Nurses from a neonatal intensive care unit  n=62  Nurses from a general pediatric ward  n=55 | Employees with no patient contact  n=46 | ─ | Complement fixation |  |
| 47 | Zając, P. W., Czarkowska-Pączek, B., & Wyczałkowska-Tomasik, A. J. J. o. O. H. (2020)(47) | March to April 2015 | 2020 | Poland | Cross-sectional | Hospitals and a family medicine outpatient clinic | Nurses from pediatrics n=30, transplantology n=30, primary health care n=30 | Women not working in health care and not working in direct contact with children n=30 | ─ | Antibody test by chemiluminescent  microparticle immunoassays using the ARCHITECT and 1000 Abbott  (Abbott Laboratories). |  |
| 48 | Zenebe, Mengistu Hailemariam, Zeleke Mekonnen, Eskindir Loha, and Elizaveta Padalko. (2021)(48) | August to October 2020 | 2021 | Ethiopia | Cross-sectional | Hospital | N total=600, Employer as Childcare worker=40, Employer as Healthcare worker=32 | - | - | ELISA |  |

**Supplementary Table 5. Funnel plot asymmetry test for publication bias**

|  | P-value | |
| --- | --- | --- |
|  | Rank correlation (Begg’s test) | Linear regression method (Egger’s test) |
| Overall prevalence | 0.06 | 0.27 |
| Overall CMV primary infection rate | 0.17 | 0.07 |
| Relative risk of CMV seropositivity | 0.76 | 0.81 |
| Relative risk CMV primary infection | 1.00 | 0.18 |
| Risk enfant | 0.27 | 0.38 |
| Marital status | 0.75 | 0.51 |
| Age | 1.00 | 0.53 |
| Race | 1.00 | 0.97 |

**Supplementary Table 6. Meta-regression**

|  | Prevalence | Primary CMV infection | Risk of CMV seropostivity | Primary CMV infection relative risk |
| --- | --- | --- | --- | --- |
| Continent |  |  |  |  |
| America | 0.7783 | Reference | Reference | Reference |
| Asia | 0.1257 | 0.5259 | NA | NA |
| Europe | 0.7387 | 0.0874 | 0.2735 | 0.1711 |
| Oceania | 0.6820 | 0.8782 | NA | NI |
| Africa | Reference | NA | NA | NA |
| Method |  |  |  |  |
| Complement fixation | 0.3518 | 0.3194 | NA | 0.1739 |
| ELISA | 0.0535 | 0.8188 | 0.3363 | 0.7559 |
| Latex agglutination | 0.1981 | 0.8585 | NA | 0.6341 |
| Not specified | 0.0232* | 0.2134 | NA | NA |
| Other | 0.3566 | 0.5290 | 0.0034* | NA |
| Restriction endonuclease | 0.8290 | 0.8541 | NA | NA |
| Anticomplement immunofluorescence assay | Reference | Reference | Reference | Reference |
| Study design (cross-sectional vs cohort) | 0.4544 | - | 0.0002* |  |
| Group (childcare worker vs health workers) | 0.0751 | 0.0735 | 0.3558 | 0.1046 |
| Study quality |  |  |  |  |
| Good | 0.0999 | 0.8597 | 0.0153* | 0.4345 |
| Poor | 0.7068 | 0.5907 | 0.0729 | NA |
| Fair | Reference | Reference | Reference | Reference |

**References (Supplementary Table 4. Characteristics of the selected articles)**

1. Adler S. The prevalence of cytomegalovirus viruria among hospitalized children and the risk of cytomegalovirus acquisition by nurses. The New England journal of medicine. 1984;310(21):1388.

2. Adler SP. Molecular epidemiology of cytomegalovirus: viral transmission among children attending a day care center, their parents, and caretakers. The Journal of pediatrics. 1988;112(3):366-72.

3. Adler S. Cytomegalovirus and child day care: Evidence for an increased infection rate among day care workers. The Pediatric infectious disease journal. 1990;9(11):862.

4. Ahlfors K, Ivarsson SA, Johnsson T, Renmarker K. Risk of cytomegalovirus infection in nurses and congenital infection in their offspring. Acta Pædiatrica. 1981;70(6):819-23.

5. Balcarek KB, Bagley R, Cloud GA, Pass RF. Cytomegalovirus infection among employees of a children's hospital: no evidence for increased risk associated with patient care. Jama. 1990;263(6):840-4.

6. Bale JF, Zimmerman B, Dawson JD, Souza IE, Petheram SJ, Murph JR. Cytomegalovirus transmission in child care homes. Archives of pediatrics & adolescent medicine. 1999;153(1):75-9.

7. Balfour CL, Balfour HH. Cytomegalovirus is not an occupational risk for nurses in renal transplant and neonatal units: results of a prospective surveillance study. Jama. 1986;256(14):1909-14.

8. BLACKMAN JA, MURPH JR, BALE JR JF. Risk of cytomegalovirus infection among educators and health care personnel serving disabled children. The Pediatric infectious disease journal. 1987;6(8):725-8.

9. Brady MT, Demmler GJ, Anderson DC. Brief Report: Cytomegalovirus Infection in Pediatric House Officers: Susceptibility to and Rate of Primary Infection. Infection Control & Hospital Epidemiology. 1987;8(8):329-32.

10. Centers—Alabama D-C. Prevalence of Cytomegalovirus Excretion from Children in Five Day-Care Centers—Alabama. Prevalence. 1985;34(4).

11. De Schryver A, Glazemakers J, De Bacquer D, De Backer G, Lust E. Risk of cytomegalovirus infection among educators and health care personnel serving mentally disabled children. Journal of Infection. 1999;38(1):36-40.

12. De Villemeur AB, Gratacap-Cavallier B, Casey R, Baccard-Longère M, Goirand L, Seigneurin J-M, Morand P. Occupational risk for cytomegalovirus, but not for parvovirus B19 in child-care personnel in France. Journal of Infection. 2011;63(6):457-67.

13. Dworsky ME, Welch K, Cassady G, Stagno S. Occupational risk for primary cytomegalovirus infection among pediatric health-care workers. New England Journal of Medicine. 1983;309(16):950-3.

14. Embil JA, Pereira LH, Manley K, MacNEIL JP. Prevalence of cytomegalovirus antibodies in the personnel of a children's hospital, Halifax, Nova Scotia. Canadian journal of public health= Revue canadienne de sante publique. 1988;79(6):455-7.

15. Ford-Jones EL, Kitai I, Davis L, Corey M, Farrell H, Petric M, Kyle I, Beach J, Yaffe B, Kelly E. Cytomegalovirus infections in Toronto child-care centers: a prospective study of viral excretion in children and seroconversion among day-care providers. The Pediatric infectious disease journal. 1996;15(6):507-14.

16. Friedman HM, Lewis MR, Nemerofsky DM, Plotkin SA. Acquisition of cytomegalovirus infection among female employees at a pediatric hospital. Pediatric infectious disease. 1984;3(3):233-5.

17. Gerberding JL. Incidence and prevalence of human immunodeficiency virus, hepatitis B virus, hepatitis C virus, and cytomegalovirus among health care personnel at risk for blood exposure: final report from a longitudinal study. Journal of infectious diseases. 1994;170(6):1410-7.

18. Hatherley LI. Prevalence of cytomegalovirus antibodies in obstetric nurses. Medical journal of Australia. 1985;142(3):186-9.

19. Hatherley LI. Is primary cytomegalovirus infection an occupational hazard for obstetric nurses? A serological study. Infection Control & Hospital Epidemiology. 1986;7(9):452-5.

20. Jackson LA, Stewart LK, Solomon SL, Boase J, Alexander ER, Heath JL, McQuillan GK, Coleman PJ, Stewart JA, Shapiro CN. Risk of infection with hepatitis A, B or C, cytomegalovirus, varicella or measles among child care providers. The Pediatric infectious disease journal. 1996;15(7):584-9.

21. Jones LA, Duke-Duncan PM, Yeager A. Cytomegaloviral infections in infant-toddler centers: centers for the developmentally delayed versus regular day care. Journal of Infectious Diseases. 1985;151(5):953-5.

22. Joseph SA, Beliveau C, Muecke CJ, Rahme E, Soto JC, Flowerdew G, Johnston L, Langille D, Gyorkos TW. Risk factors for cytomegalovirus seropositivity in a population of day care educators in Montreal, Canada. Occupational medicine. 2005;55(7):564-7.

23. Kiss P, De Bacquer D, Sergooris L, De Meester M, Vanhoorne M. Cytomegalovirus infection: an occupational hazard to kindergarten teachers working with children aged 2.5–6 years. International journal of occupational and environmental health. 2002;8(2):79-86.

24. Lamarre V, Gilbert N, Rousseau C, Gyorkos TW, Fraser W. Seroconversion for cytomegalovirus infection in a cohort of pregnant women in Québec, 2010–2013. Epidemiology & Infection. 2016;144(8):1701-9.

25. Lepage N, Leroyer A, Cherot-Kornobis N, Lartigau I, Miczek S, Sobaszek A. Cytomegalovirus seroprevalence in exposed and unexposed populations of hospital employees. Eur J Clin Microbiol Infect Dis. 2011;30(1):65-70.

26. Leroux M, Reinert P, Boucher J, Lasry S. Diffusion du cytomégalovirus en crèche. Journal de Pédiatrie et de Puériculture. 2002;15(5):299-301.

27. Lipscomb JA, Linnemann Jr CC, Hurst PF, Myers MG, Stringer W, Moore P, Hammond J. Prevalence of cytomegalovirus antibody in nursing personnel. Infection Control. 1984:513-8.

28. Morgan MA, Khalifa NA, Sherif A, Rasslan L. Prevalence of cytomegalovirus (CMV) infection among neonatal intensive care unit (NICU) and healthcare workers. The Egyptian journal of immunology. 2003;10(2):1-8.

29. Murph JR, Bale Jr JF, Murray JC, Stinski MF, Perlman S. Cytomegalovirus transmission in a Midwest day care center: possible relationship to child care practices. The Journal of pediatrics. 1986;109(1):35-9.

30. Murph JR, Bale JF, Perlman S, Swack NS, Smith FG. 566 THE PREVALENCE OF CYTOMEGALOVIRUS INFECTION IN MIDWEST DAY CAKE CENTER. Pediatr Res. 1985;19(4):205-.

31. Murph JR, Baron JC, Brown CK, Ebelhack CL, Bale JF. The occupational risk of cytomegalovirus infection among day-care providers. Jama. 1991;265(5):603-8.

32. Nelson D, Peckham C, Pearl K, Chin K, Garrett A, Warren D. Cytomegalovirus infection in day nurseries. Archives of disease in childhood. 1987;62(4):329-32.

33. Okwori A, Olabode A, Emumwen E, Echeonwu G, Lugos M, Okpe E, Okopi J, Adetunji J. Sero-epidemiological survey of human cytomegalo virus infection among expectant mothers in Bida, Nigeria. 2008.

34. Pass RF, Hutto C, Lyon M, Cloud GJTPidj. Increased rate of cytomegalovirus infection among day care center workers. 1990;9(7):465-70.

35. Sobaszek A, Fantoni-Quinton S, Frimat P, Leroyer A, Laynat A, Edme J-L. Prevalence of cytomegalovirus infection among health care workers in pediatric and immunosuppressed adult units. Journal of occupational and environmental medicine. 2000;42(11):1109-14.

36. Soto JC, Delage G. Cytomegalovirus infection as an occupational hazard among women employed in day-care centers. Pediatrics. 1994;94(6):1031-.

37. Stelma F, Smismans A, Goossens V, Bruggeman C, Hoebe C. Occupational risk of human Cytomegalovirus and Parvovirus B19 infection in female day care personnel in the Netherlands; a study based on seroprevalence. Eur J Clin Microbiol Infect Dis. 2009;28(4):393-7.

38. Stranzinger J, Kindel J, Henning M, Wendeler D, Nienhaus A. Prevalence of CMV infection among staff in a metropolitan children’s hospital–occupational health screening findings. GMS hygiene and infection control. 2016;11.

39. Stranzinger J, Kozak A, Schilgen B, Paris D, Nießen T, Schmidt L, Wille A, Wagner NL, Nienhaus A. Are female daycare workers at greater risk of cytomegalovirus infection? A secondary data analysis of CMV seroprevalence between 2010 and 2013 in Hamburg, Germany. GMS hygiene and infection control. 2016;11.

40. Takao M, Yoshioka N, Hagiya H, Deguchi M, Kagita M, Tsukamoto H, Hidaka Y, Tomono K, Tobe T. Risk for the occupational infection by cytomegalovirus among health-care workers. Journal of infection and chemotherapy : official journal of the Japan Society of Chemotherapy. 2020;26(7):681-4.

41. TOLKOFF-RUBIN NE, RUBIN RH, KELLER EE, BAKER GP, STEWART JA, HIRSCH MS. Cytomegalovirus infection in dialysis patients and personnel. Annals of internal medicine. 1978;89(5_Part_1):625-8.

42. Uslu H, Uyanik MH, Akpinar RB, Celebioglu A. Seroprevalence of rubella and cytomegalovirus in nursing students. Pakistan Journal of Medical Sciences. 2010;26(3).

43. van Rijckevorsel GGC, Bovée LPJ, Damen M, Sonder GJ, van der Loeff MFS, van den Hoek A. Increased seroprevalence of IgG-class antibodies against cytomegalovirus, parvovirus B19, and varicella-zoster virus in women working in child day care. BMC public health. 2012;12(1):475.

44. Volpi A, Pica F, Cauletti M, Pana A, Rocchi G. Cytomegalovirus infection in day care centers in Rome, Italy: viral excretion in children and occupational risk among workers. Journal of medical virology. 1988;26(2):119-25.

45. Wicker S. Viral infections: occupational risk for pregnant health-care personnel? Procedia in Vaccinology. 2012;6:156-8.

46. Yeager AS. Longitudinal, serological study of cytomegalovirus infections in nurses and in personnel without patient contact. Journal of clinical microbiology. 1975;2(5):448-52.

47. Zając PW, Czarkowska-Pączek B, Wyczałkowska-Tomasik A. Prevalence and molecular epidemiology of CMV and EBV among nurses working in pediatrics, transplantology, and primary health care. Journal of occupational health. 2020;62(1):e12112.

48. Zenebe MH, Mekonnen Z, Loha E, Padalko EJBo. Seroprevalence and associated factors of maternal cytomegalovirus in Southern Ethiopia: a cross-sectional study. 2021;11(10):e051390.
